# In vitro derivation of midbrain dopaminergic neurons from porcine embryonic stem cells in multi-dimensional conditions

**DOI:** 10.1186/s13287-025-04693-9

**Published:** 2025-11-05

**Authors:** Hyerin Choi, Dongjin Oh, Ali Jawad, Zheng Haomiao, Jaehyung Ham, Juyoung Heo, Aram Oh, Huijin Jin, Jaehyeok Seo, Byoung Chol Oh, Sang-Hwan Hyun

**Affiliations:** 1 Veterinary Medical Center, College of Veterinary Medicine, Laboratory of Veterinary Embryology and Biotechnology (VETEMBIO), Chungbuk National University, Cheongju, Republic of Korea; 2 Institute of Stem Cell and Regenerative Medicine (ISCRM), Chungbuk National University, Cheongju, Republic of Korea; 3 Vet-ICT Convergence Education and Research Center (VICERC), Chungbuk National University, Cheongju, Republic of Korea; 4 Chungbuk National University Hospital, Cheongju, Republic of Korea; 5 Department of Neurology, Institute for Cell Engineering, School of Medicine, Johns Hopkins Medicine, Baltimore, ML USA; 6 Department of Plastic and Reconstructive Surgery, Johns Hopkins University School of Medicine, Baltimore, MD USA; 7 Laboratory of Veterinary Embryology and Biotechnology (VETEMBIO), College of Veterinary Medicine, Chungbuk National University, 1 Chungdae-ro, Seowon-gu, Cheongju, 28644 Republic of Korea

**Keywords:** Porcine, Embryonic stem cells, Midbrain, Dopaminergic neuron, Organoid

## Abstract

**Background:**

. Porcine pluripotent stem cells hold significant promise as a large-animal model for human neurodevelopment and disease modeling. However, efficient protocols for their directed differentiation into midbrain dopaminergic (mDA) neurons and the generation of 3D midbrain-like organoids remain limited. This study aimed to establish species-optimized conditions for the derivation of functional mDA neurons from porcine embryonic stem cells (pESCs) under both two- and three-dimensional environments.

**Methods:**

. In vitro fertilization (IVF)- and parthenogenetic activation (PA)-derived pESCs were subjected to neural induction using stepwise exposure to dual SMAD inhibition, SHH, CHIR99021, and FGF8. For monolayer culture, adherent monolayers were differentiated on Matrigel- or poly-L-ornithine/laminin I/fibronectin-coated surfaces. For 3D culture, porcine midbrain-like organoids (pMLOs) were formed under low-adhesion conditions. Functional and molecular characterization was performed via immunofluorescence, patch-clamp electrophysiology, microelectrode array (MEA) recordings, dopamine ELISA, and RNA sequencing.

**Results:**

. Porcine embryonic stem cells required higher SHH and GSK3 inhibition thresholds to efficiently induce FOXA2⁺ ventral midbrain progenitors, revealing a species-specific divergence from human protocols. IVF-derived pESCs showed markedly enhanced dopaminergic differentiation and functional maturation compared to PA-derived pESCs. Notably, in 3D culture, neuroepithelial structures emerged as early as day 5, and functionally mature mDA neurons—confirmed by TH expression, dopamine release, and spontaneous synaptic activity—were detected by day 28. This timeline represents an accelerated maturation relative to comparable human mDA differentiation systems, where functional properties typically arise after day 35. Single-cell RNA sequencing further delineated dynamic dopaminergic lineage trajectories and revealed porcine-specific gene expression patterns associated with early mDA identity acquisition.

**Conclusions:**

. This study presents the first robust platform for generating functionally validated mDA neurons and midbrain-like organoids from porcine stem cells. The findings highlight species-specific signaling dynamics and establish porcine in vitro models as scalable, translational systems for investigating neurodevelopment and disease mechanisms.

**Supplementary Information:**

The online version contains supplementary material available at 10.1186/s13287-025-04693-9.

## Background

The human brain has the longest and slowest development process among organs and features, a complex structure [1, 2]. Animal models play an indispensable role in neuroscience research, providing critical insights into disease mechanisms, drug development, and data that cannot be obtained from human studies [3]. Comparative studies of human brain structures have primarily utilized rodents and non-human primates (NHPs) [4, 5]. On the other hand, few comparisons have been made with large mammals such as pigs, cows, sheep, and horses. The magnetic resonance imaging studies have revealed similarities in brain development between humans and pigs [6, 7]. Pigs have a gyrencephalic brain and are less complex in structure than NHPs, aligning with the “replacement” principle of animal research [7]. Additionally, the postnatal brain growth in pigs closely resembles that of human neonates [8], while their gestation period is significantly shorter (~ 114 days vs. ~280 days) [9]. This contrast highlights species-specific developmental pacing and supports the use of pigs as an efficient large-animal model for early brain development studies.

Parkinson’s disease (PD) is a neurodegenerative disorder that primarily affects middle-aged and older adults, and survival rates for PD patients are lower than the general population [10, 11]. PD is characterized by the localized degeneration of midbrain dopaminergic (mDA) neurons in the substantia nigra (SN), making it a key target for stem cell-based therapies [12]. The SN, a midbrain dopaminergic nucleus, is crucial for modulating motor movement and reward function within the basal ganglia circuitry [13]. Pigs are valuable for modeling human PD because their SN is anatomically more similar to humans than rodents [14–16]. For these reasons, genetically modified pig PD models have been generated using somatic cell nuclear transfer and the CRISPR/Cas9 system [17–19]. Nevertheless, the porcine PD models did not show the obvious neuronal loss and behavioral abnormalities that can be observed in human PD. Additionally, efforts to enhance in vivo models are time-consuming and expensive. Therefore, a comprehensive understanding of the porcine nervous system is essential to create an effective in vivo model.

Recent technological advances in the stem cell field have resulted in organoids, which are self-organized 3D tissues. The brain organoid is particularly valuable as it provides the opportunity to understand complex biological processes in a physiologically relevant context, and it facilitates advancements in translational applications [20–24]. Despite these advances, current in vitro studies on the porcine nervous system are limited to the early stages of neural differentiation [25–27].

Our study aims to derive ventral midbrain (VM) progenitors from porcine embryonic stem cells (pESCs) using a floor plate transition-based method and to elucidate their maturation capacity into mDA neurons under monolayer culture conditions. Additionally, we adapt this protocol to generate midbrain-like organoids in a three-dimensional culture environment.

## Methods

### Maintenance and culture of pESCs

The pESC lines (PA_3_pESC_FIW and IVF_1_pESC_FIW) used in this study were previously established from IVF and PA blastocysts, as described in [28]. Briefly, blastocysts derived from PA or IVF were cultured on mitotically inactivated mouse embryonic fibroblasts using a FIW medium under 5% CO_2_ at 37 ℃ with daily medium exchange. The basal medium of the FIW was composed of DMEM/F12 (Thermo Fisher Scientific, #21331020), 10% knockout serum replacement (Thermo Fisher Scientific, #10828028), 1× non-essential amino acids (Thermo Fisher Scientific, #11140050), 0.05 mM β-mercaptoethanol (Thermo Fisher Scientific, #21985023), and 1% antibiotic-antimycotic (Thermo Fisher Scientific, #15240062). To prepare the FIW medium, 10 ng/mL FGF2 (PeproTech, #100-18B), 1.5 µM IWR-1 (I0161), and 0.3 µM WH-4-023 (Selleckchem, S7565) were added to the basal medium. pESC-FIW cells were dissociated using TrypLE™ Express Enzyme (Gibco, 12605010) and passaged every 3–4 days at a ratio of 1:5 to 1:10 in the presence of 10 µM Y-27,632 (STEM CELL, #72304), which was added 24 h after passage. Established pESC lines were characterized by the expression of pluripotency markers (e.g., OCT4, SOX2, NANOG) and confirmed for their differentiation potential. For more detailed information on the derivation and characterization of these lines, see [28].

### Induction of mDA neurons from pESCs in monolayer conditions

mDA neurons were differentiated from PA- and IVF-pESCs using a floor-plate-based neural induction protocol [29]. Initially, two pESC lines were seeded at a density of 0.5 × 10⁴ cells/cm² on Matrigel (Corning, #CLS354277) in FIW medium supplemented with Y-27,632 and cultured for 48 h. On day 0 of differentiation, the medium was changed to N2B27, which is composed of a 1:1 mixture of DMEM/F12 and Neurobasal medium (Thermo Fisher Scientific, #21331020 and #21103049), along with 1% N2 supplement (Thermo Fisher Scientific, #17502048), 0.5% B27 supplement (Thermo Fisher Scientific, #12587010), 1x Glutamax (Thermo Fisher Scientific, #35050061), and 1x Antibiotic-Antimycotic (Thermo Fisher Scientific, #15240062). This medium was further supplemented with 10 µM SB431542 (MCE, #HY-10431), 100 ng/ml Noggin (Peprotech, #120–10 C-100UG), 500 ng/ml SHH-C24II (Miltenyi, #130-095-727), and 1.5 µM CHIR99021 (CH) (Selleckchem, #S1263). Medium changes were conducted on days 2, 4, and 7. On day 9, the medium was switched to N2B27 with 100 ng/ml FGF8 (Miltenyi, #130-095-738) for 2 days. On day 11, the differentiated cells were dissociated using TrypLE™ Express Enzyme and replated on Matrigel-coated plates at a density of 8.0 × 10⁵ cells/cm². The cells were then cultured in B27 medium (Neurobasal, 2% B27 supplement, 1x Glutamax, 1x Antibiotic-Antimycotic) supplemented with 100 ng/ml FGF8b, 20 ng/ml BDNF (PeproTech, #450-02-10UG), and 0.2 mM ascorbic acid (AA) (#A4544). Y-27,632 was added for the first 48 h after replating, with the medium refreshed every 2–3 days. From day 16 onward, for terminal differentiation, PA- and IVF-derived cells were replated onto plates coated with poly-L-ornithine (1.5 µg/mL), laminin I (1 µg/mL), and fibronectin (2 µg/mL) at a density of 3.4 × 10⁵ cells/cm² using TrypLE™ Express Enzyme. These cells were cultured in B27 medium supplemented with 20 ng/ml BDNF, 10 ng/ml GDNF (R&D Systems, #212-GD-010), 0.2 mM ascorbic acid, 0.5 mM db-cAMP (STEM CELL, #73886), and 10 µM DAPT (MCE, HY-13027), with medium changes every other day until they were ready for analysis.

### Immunofluorescence analysis

The differentiated cells cultured under monolayer conditions were first washed with DPBS (Welgene, LB 001–02) and then fixed with 4% paraformaldehyde at room temperature for 10 min. After three washes with DPBS, the cells were permeabilized in 0.5% Triton X-100 for 10 min, and subsequently blocked with blocking buffer (CST, #12411) for 30 min. The cells were incubated overnight at 4 °C with primary antibodies diluted in blocking buffer. Following this, they were washed three times with DPBS for 5 min each. The secondary antibodies were then diluted, incubated with DPBS at room temperature for 1 h, and washed three times with DPBS for 5 min each. Finally, the cells were stained with Hoechst (Invitrogen, H3570) for 10 min. The list of antibodies used is provided in Supplementary Information Table S1.

### qRT-PCR

All samples were washed twice in Dulbecco’s PBS and stored at − 80 ℃ until mRNA extraction. Total RNA was extracted using the TRIzol reagent (TaKaRa Bio, Inc., Otsu, Japan), and complementary DNA (cDNA) was synthesized using a reverse transcription master mix (Elpis Bio, Inc., Chungcheongnam-do, Daejeon, Republic of Korea) according to the manufacturer’s instructions. For qRT-PCR, the synthesized cDNA, 2× SYBR Premix Ex Taq (TaKaRa Bio, Inc.), and 10 pmol of specific primers (Macrogen) were added to prepare the PCR mixture. All primer sequences used in this study are listed in Table S2. qRT-PCR was performed using a CFX96 Touch Real-Time PCR Detection System (Bio-Rad, Hercules, CA, United States). The reactions were performed as follows: 40 cycles of denaturation at 95 ℃ for 30 s, annealing at 58 ℃ for 15 s, and extension at 72 ℃ for 30 s. Relative quantification was performed using threshold cycle (Ct)-based methodologies at a constant fluorescence intensity. The relative mRNA expression (R) was calculated using the equation *R* = 2^−(*Ctsample* − *Ctcontrol*)^. The R-values obtained for each gene were normalized to *RN18S*.

### Whole cell patch-clamp recording of pESCs-derived DA neurons

Whole-cell recordings were made on random cells in DA neurons from PA- and IVF-pESCs. For each group, neurons were derived from the same culture batch. The recordings were conducted in artificial cerebrospinal fluid (ACSF in mM: 124 NaCl, 2.5 KCl, 1.2 NaH_2_PO_4_, 24 NaHCO_3_, 5 HEPES, 12.5 glucose, 2 MgSO_4_, and 2 CaCl_2_, pH 7.4) using Multiclamp 700B amplifier (Molecular Devices, Sunnyvale, CA), DigiDATA (Molecular Devices), and pClamp software (version 10.6. Molecular Device) at a 10 kHz sampling rate and a 2 kHz filtering rate. The patch-pipettes (4–8 MΩ) were filled with internal solution (in mM: 120 K-gluconate, 10 KCl, 2 Mg-ATP, 0.5 Na-GTP, 0.5 EGTA, 20 HEPES, and 10 phosphocreatine, pH 7.3). After making whole cell recording configuration, the resting membrane potential (RMP) was measured in I = 0 configuration. The membrane capacitance (Cm), membrane resistance (Rm), and access resistance (Ra) of the cells were calculated from recordings under voltage-clamp configuration where the cells were held at − 60 mV. The ability to fire action potential(s) (APs) was recorded under current-clamp mode by injecting 20 pA current steps for 1 s that modulated membrane potentials from hyperpolarizing 0 pA to + 200 pA. After making whole cell recording configuration, the spontaneous excitatory postsynaptic currents (sEPSC) were recorded for 5 min at -60 mV using gap free protocol.

### Transcriptome analysis

Total RNA concentration was calculated by Quant-IT RiboGreen (Invitrogen, #R11490). To assess the integrity of the total RNA, samples are run on the TapeStation RNA screentape (Agilent, #5067–5576). Only high-quality RNA preparations, with an RIN greater than 7.0, were used for RNA library construction.

A library was independently prepared with 1 µg of total RNA for each sample by Illumina TruSeq Stranded mRNA Sample Prep Kit (Illumina, Inc., San Diego, CA, USA, #20020595). The first step in the workflow involves purifying the poly-A containing mRNA molecules using poly‐T‐attached magnetic beads. Following purification, the mRNA is fragmented into small pieces using divalent cations under elevated temperature. The cleaved RNA fragments are copied into first strand cDNA using SuperScript II reverse transcriptase (Invitrogen, #18064014) and random primers. This is followed by second strand cDNA synthesis using DNA Polymerase I, RNase H and dUTP. These cDNA fragments then go through an end repair process, the addition of a single ‘A’ base, and then ligation of the adapters. The products are then purified and enriched with PCR to create the final cDNA library.

The libraries were quantified using KAPA Library Quantification kits for Illumina Sequencing platforms according to the qPCR Quantification Protocol Guide (KAPA BIOSYSTEMS, #KK4854) and qualified using the TapeStation D1000 ScreenTape (Agilent Technologies, # 5067–5582). Indexed libraries were then submitted to an Illumina NovaSeq (Illumina, Inc., San Diego, CA, USA), and the paired-end (2 × 100 bp) sequencing was performed by the Macrogen Incorporated.

### Data processing and analysis

Paired-end sequencing reads were generated on the Illumina sequencing NovaSeq platform. Before starting analysis, Trimmomatic v0.38 was used to remove adapter sequences and trim bases with poor base quality. The cleaned reads were aligned to the *Sus scrofa (Sscrofa11.1*) using HISAT v2.1.0 [30]. The reference genome sequence and gene annotation data were downloaded from NCBI Genome assembly and NCBI RefSeq database respectively. Aligned data (SAM file format) were sorted and indexed using SAMtools v 1.9. After alignment, the transcripts were assembled and quantified using StringTie v2.1.3b [31, 32]. Gene-level and Transcript-level quantification were calculated as raw read count, FPKM (Fragments Per Kilobase of transcript per Million mapped reads) and TPM (Transcripts Per Million).

### Differential gene expression analysis

Statistical analyses of differential gene expression were performed by DESeq2 v 1.38.3 [33] using raw counts as input. In the QC step, genes with non-zero counts in all samples were selected. PCA (Principal component analysis) and MDS (Multidimensional scaling) plot were generated to confirm the similarity of expression between samples. Filtered data set was applied with RLE normalization to correct the variation of library sizes among samples. Statistical significance of differential expression gene was determined using DESeq2 nbinom WaldTest [33]. Fold change and p-value were extracted from the result of WaldTest. All p-values are adjusted by Benjamini-Hochberg algorithm to control false discovery rate. Significant gene list was filtered by |fold change|≥2 & raw p-value < 0.05. Hierarchical clustering on rlog transformed values for significant genes was performed with these parameters (distance metric = Euclidean distance, linkage method = complete). Gene-enrichment and functional annotation analysis for significant genes were carried out using gProfiler [34] (https://biit.cs.ut.ee/gprofiler/orth) against GO (Gene Ontology) database and using in-house KEGG Viewer script against KEGG pathway database (http://www.genome.jp/kegg/pathway.html). Adjusted p-values reported from gProfiler were derived using one-sided hypergeometric test and corrected by Benjamini-Hochberg method. Adjusted p-values reported from KEGG viewer result were derived using two-sided modified Fisher’s exact test and corrected by Benjamini-Hochberg method. All data analysis and visualization of differentially expressed genes was conducted using R 4.2.2 (www.r-project.org).

### Generation of porcine midbrain-like organoids

IVF_1_ pESC_FIW cells were used for the generation of porcine midbrain-like organoids. The pESCs were dissociated using TrypLE™ Express Enzyme for 3 min at 37 ℃. Following this, cells were plated in pre-coated plates with 2% gelatin to remove MEF cells for 15 min at 37 ℃. The cells were then collected and counted using a hemocytometer. A total 2,500 single cells were plated in each well of a 96-well ultra-low attachment U-bottom plate (Corning, #7007) with mTeSR™ Plus (STEM CELL, #100–0276) supplemented with 10 µM Y-27,632 to form embryoid bodies (EBs). After 2 days in culture, the EBs were transferred to a 6-well ultra-low attachment plate (Corning, #3471) containing N2B27 medium supplemented with 10 µM SB431542, 100 ng/ml Noggin, 500 ng/ml SHH-C24II, and 1.5 µM CHIR99021 (day 0). The medium was changed on day 3. On day 5, the medium was replaced with N2B27 containing 100 ng/ml FGF8. After an additional 3 days, the medium composition was switched to B27 medium supplemented with 100 ng/ml FGF8, 20 ng/ml BDNF, and 0.2 mM AA. On day 13, EBs were embedded in 30 µl droplet of Matrigel for 30 min at 37 ℃. For terminal differentiation, the organoids were cultured in B27 medium supplemented with 20 ng/ml BDNF, 10 ng/ml GDNF, 0.2 mM ascorbic acid, 0.5 mM db-cAMP, and 10 µM DAPT and incubated on an orbital shaker. Medium changes were performed every three days until the organoids were ready for analysis.

### Organoid cryosectioning and immunofluorescence

Porcine midbrain-like organoids were washed once in PBS and fixed in 4% paraformaldehyde for 5 h at room temperature on an orbital shaker. They were then washed in PBS three times for 5 min each. The organoids were allowed to sink in 30% sucrose overnight at 4 ℃. The sucrose solution was replaced with a 1:1 mixture of OCT and 30% w/v sucrose for 6 h, and then the organoids were transferred to a cryomold and filled with OCT. The embedded tissue was frozen on dry ice and cryosectioned at 10 μm. For immunohistochemistry, the sections were washed in PBS three times for 5 min each and fixed in 4% paraformaldehyde for 10 min at room temperature. After this, the sections were washed again in PBS three times for 5 min each. They were then blocked and permeabilized in 0.3% v/v Triton X-100 in blocking buffer for 1 h at room temperature. Each slide was incubated overnight at 4 ℃ with a primary antibody solution. The following day, sections were washed in PBS three times for 5 min and then incubated for 1 h with the appropriate secondary antibodies (diluted 1:400). After wahsing with PBS three times for 5 min each, the sections were stained with Hoechst for 10 min. Each slide was mounted using antifade (Thermo Fisher Scientific, #P36934) and coverslipped. A list of antibodies is provided in Table S2.

### Microelectrode array (MEA) recordings

MEA plates (6-well format, Axion BioSystems) were prepared following the Axion BioSystems Maestro Plating Protocol [35]. Wells were pre-coated with 0.1% polyethylenimine in borate buffer (pH 8.4) to facilitate organoid attachment. The plates were incubated at room temperature for 1 h, washed four times with sterile water, and air-dried overnight under sterile conditions. Prior to organoid seeding, the wells were coated with laminin (10 µg/mL in maintenance medium) and incubated for 1 h at 37 °C. Organoids were carefully transferred into the pre-coated wells and positioned over the MEA electrodes. Following an initial attachment period overnight at 37 °C in a 5% CO₂ incubator, additional maintenance medium was added to fully submerge the organoids. MEA recordings were performed using the Axion Maestro Pro system with AxIS Navigator software. Brain organoid activity was assessed by analyzing spike detection and burst patterns according to the Axion BioSystems guidelines. Only wells with properly attached organoids and adequate electrode coverage were included in the analysis.

### Single-cell RNA sequencing and data analysis

Single-cell RNA sequencing (scRNA-seq) libraries were prepared using the Chromium X platform following the 10x Genomics GEM-X Single Cell 3’ Reagent Kit v4 protocol (CG000731). Cell suspensions were diluted in PBS to achieve a target of 10,000 cells per sample, mixed with a master mix, and loaded into a Chromium GEM-X Single Cell 3’ Chip v4, along with Single Cell 3’ v4 Gel beads and Partitioning Oil. RNA transcripts from individual cells were uniquely barcoded and reverse-transcribed within droplets. The resulting cDNA molecules were pooled and subjected to end repair, A-tailing, and adapter ligation. The final cDNA libraries were purified and enriched via PCR. Library quality was assessed using qPCR (KAPA Biosystems) and the Agilent Technologies 4200 TapeStation. The sequencing was performed on the Illumina NovaSeq platform according to the manufacturer’s protocol.

Raw sequencing data were processed using Cell Ranger v8.0.1 (10x Genomics; https://support.10xgenomics.com). Briefly, BCL files generated from the NovaSeq platform were demultiplexed into FASTQ files using ‘cellranger mkfastq’. These files were then analyzed using ‘cellranger count’, which included alignment to the Sus scrofa reference genome (Sscrofa11.1), quantification of gene expression based on unique molecular identifiers and cell barcodes, cell clustering, and differential gene expression analysis. Data from multiple sequencing runs of the same library were aggregated using ‘cellranger aggr’.

The raw count matrices obtained from ‘cellranger aggr’ were imported into Seurat v4.3.0 for downstream analysis. Genes detected in fewer than 3 cells and cells expressing fewer than 200 genes were removed. To eliminate multiplets and low-quality cells, cells with more than 7,000 detected genes or mitochondrial gene content exceeding 10% were filtered out. After quality control, 26,655 genes across 103,310 cells were retained for further analysis. Gene expression values were normalized and scaled, followed by PCA for dimensionality reduction. Clustering was performed, and Uniform Manifold Approximation and Projection (UMAP) was used for visualization. Differentially expressed genes (DEGs) were identified using the Wilcoxon rank-sum test, with a minimum percentage threshold of 0.25 and a log fold change threshold of 0.25. Functional enrichment analysis of significant gene lists was performed using g: Profiler (https://biit.cs.ut.ee/gprofiler/).

### Statistical analysis

Statistical analyses were performed using the Prism software (version 8.0; GraphPad Software, San Diego, CA, USA). Results are expressed as mean ± standard error of the mean (SEM). Experiments were repeated at least three times unless a different number of repeats was specified in the legend. Statistical analyses were performed using an unpaired two-tailed Student’s *t*-test or ANOVA. *p* < 0.05 was considered statistically significant. The statistical methods, p-values, and sample numbers are indicated in the figure legends.

## Results

### Development of an optimal protocol to derive VM progenitors from pESCs

**Fig. 1:**
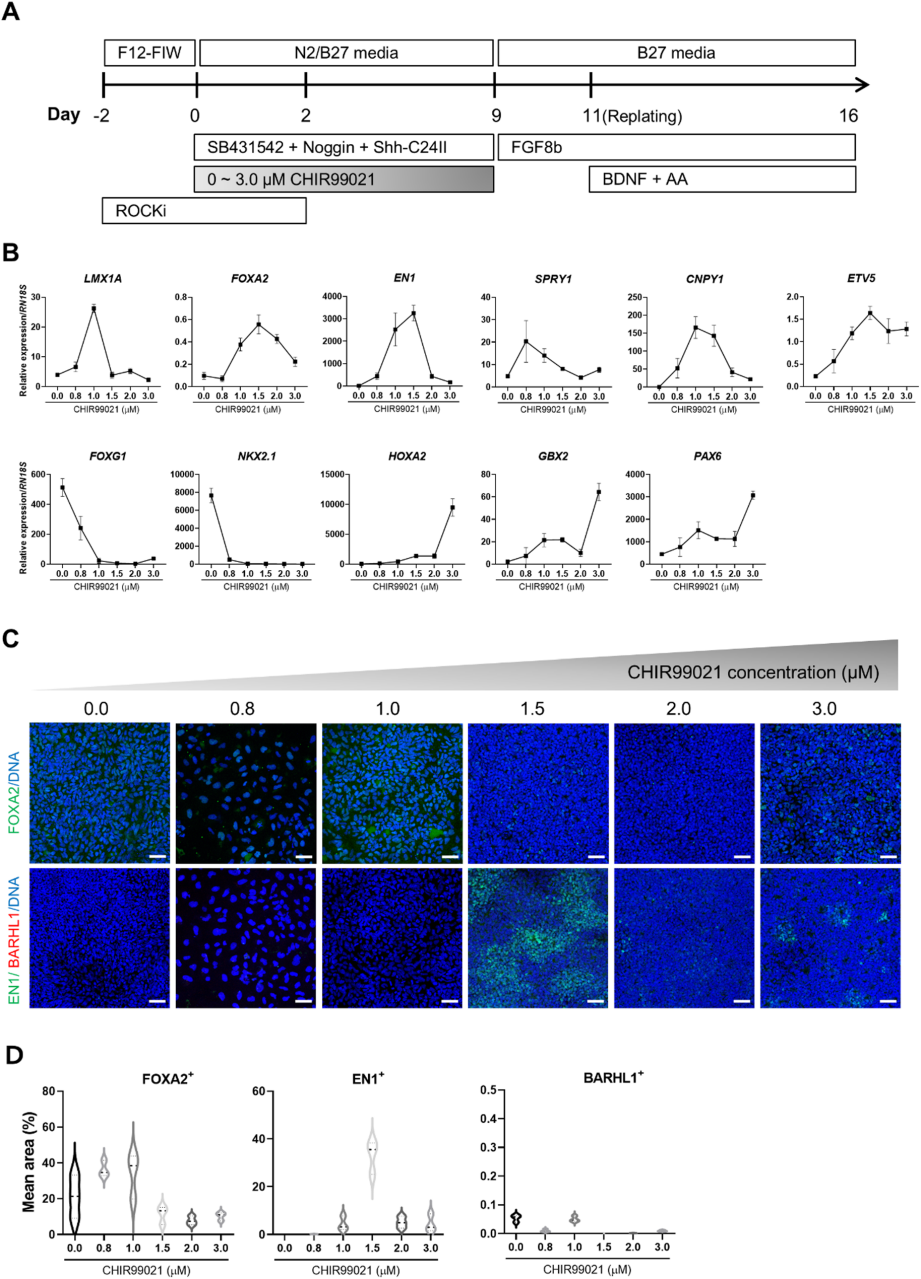
Ventral midbrain (VM) patterning of porcine embryonic stem cells according to the concentration of CHIR99021 in 2D condition. (A) Schematic overview of the differentiation protocol for VM patterning with growth factors, small molecules, and culture medium. (B) RT–qPCR analysis of differentiated cells according to CHIR99021 concentration at day 16 (*n* = 3 independent experiments). Results are given as fold change over undifferentiated pESCs. (C and D) Immunostaining and quantification of day 16 differentiated cells positive for FOXA2, BARHL1, and EN1. Scale bar, 20 μm

We adapted a floor-plate transition-based method [28] to derive VM progenitors from pESCs (Figs. 1 A and S1A). This differentiation protocol includes ventralization with SHH and caudalization using GSK3 inhibitor CH, and FGF8. As in humans, the absence of GSK3 inhibition during porcine VM patterning resulted in the upregulation of forebrain-related genes (*FOXG1* and *NKX2.1*) and high concentration of CH (3.0 µΜ) promoted expression of hindbrain markers (*HOXA2* and *GBX2*) (Fig. 1B). On the other hand, 1.5 µΜ CH supplementation caused progenitors toward midbrain fate based on high transcript levels of caudal VM marker genes (*EN1*, *SPRY1*, *CNPY1*, and *ETV5*) (Fig. 1B) and the presence of EN1-positive cells at day 16 (Figs. 1C and S1B). Studies in mice revealed that mDA lineage, identified by EN1 and CNPY1 expression, is abundant in the caudal midbrain [36–38]. Therefore, we selected 1.5 µM CH as the optimal concentration for establishing porcine VM progenitors.

To address the low population of FOXA2^+^ cells (approximately 11.3%) observed in our results (Fig. 1D), we sought to improve the culture conditions for VM progenitors by replacing Matrigel with Laminin-111 (LN111). LN111 is a physiologically relevant extracellular matrix component found in the developing brain that supports the attachment of differentiating neural progenitors but not pluripotent stem cells [38]. We cultured pESCs on either Matrigel or full-length LN111 until day 26 for differentiation (Figure S2B). By day 11, the cell yield had increased approximately 400-fold compared to day 0 in both conditions, although a low cell population was observed in the LN111 condition on day 7 (Figure S2B). qRT-PCR analysis revealed that the LN111 condition did not enhance the expression of VM marker genes (*LMX1A*, *FOXA2*, and *OTX2*), nor did it reduce the expression of the lateral marker PAX6 (Figure S2C). On day 16, both conditions showed a high purity of OTX2^+^ cells, with very few FOXA2^+^ cells present (Figure S2C). We continued to maintain the differentiated cells until day 26, confirming the presence of mature DA markers TH, EN1, and MAP2, with no expression of the glial marker GFAP (Figure S2E). Thus, LN111, which is known to support human VM progenitors in vitro, demonstrated a similar effect on porcine VM patterning as Matrigel.

**Fig. 2:**
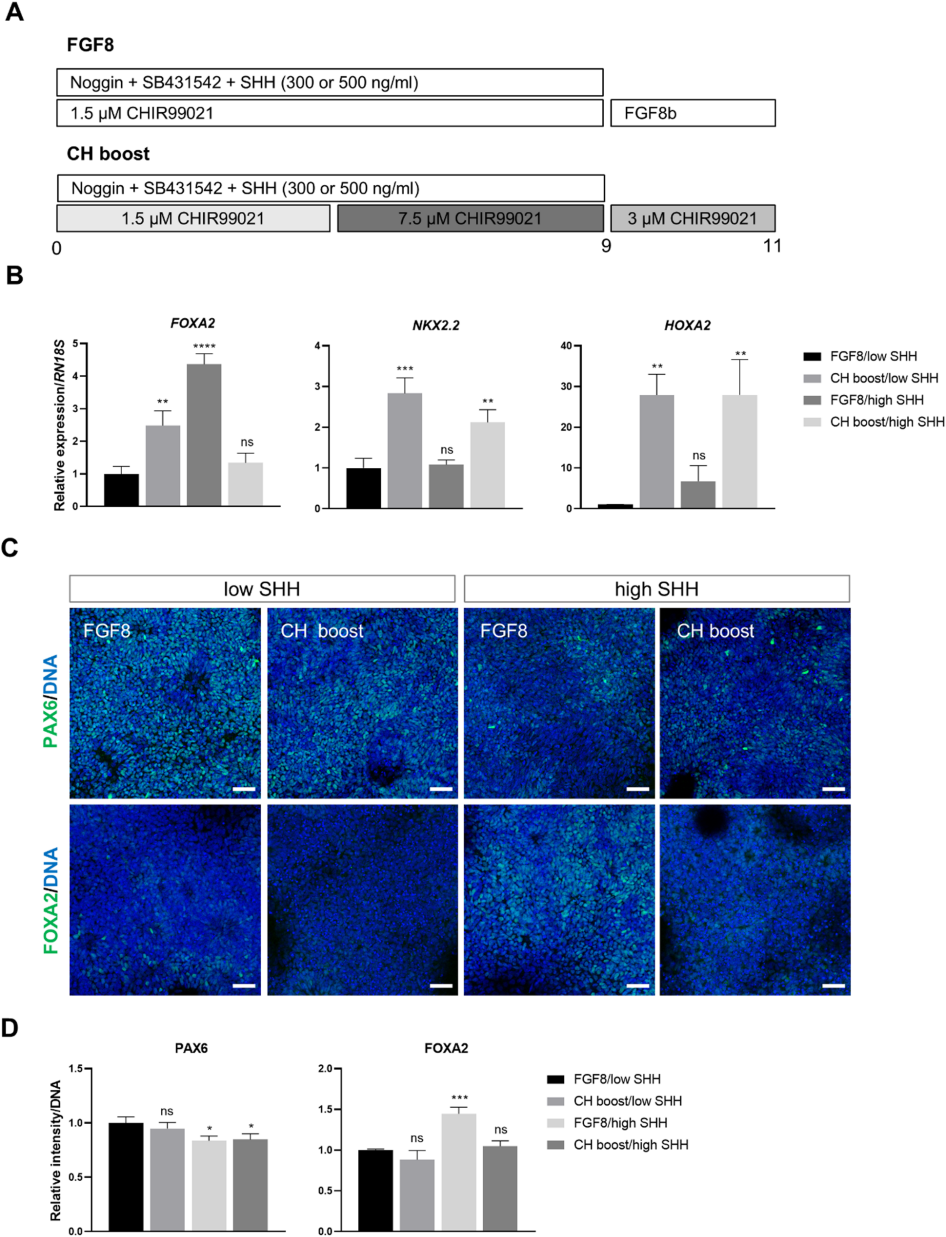
Effects of SHH concentration and CH boost on the VM patterning. (A) Schematic illustration of the FGF8 or CH boost culture conditions tested. (B) RT-qPCR analysis of differentiated cells at day 11. (C) Immunostaining of PAX6 and FOXA2 at day 11. Scale bar, 20 μm. (D) Relative intensity of PAX6^+^ cells and FOXA2^+^ cells. ns, not significant; * *p* < 0.05, ** *p* < 0.01, *** *p* < 0.001, **** *p* < 0.0001

To enhance the expression efficiency of VM progenitor markers, we introduced a bi-phasic WNT activation protocol [39]. This method establishes midbrain/hindbrain identity by initially using a low concentration of CH, followed by a higher concentration to direct midbrain fate, avoiding the use of extrinsic FGF8. As a result, we observed robust and consistent induction of the midbrain marker EN1 by day 11, surpassing previous protocols [30]. We applied CH concentrations of 1.5, 7.5, and 3.0 µM without FGF8 for 11 days (‘CH boost’) and administered SHH at either 300 ng/ml (low) or 500 ng/ml (high) (Fig. 2A). qRT-PCR analysis indicated that the highest expression of *FOXA2* occurred in the ‘FGF8/high SHH’ condition (Fig. 2B). In contrast, both CH boost conditions led to the upregulation of the diencephalic marker *NKX2.2* and the hindbrain marker *HOXA2*. Immunofluorescence results showed that the high SHH condition decreased the number of PAX6^+^ cells (Figs. 2C and D), while FOXA2 expression improved only under the FGF8/high SHH condition. These findings suggest that treatment with FGF8 and high SHH enhances the derivation of porcine VM progenitor cells by downregulating PAX6 and upregulating FOXA2.

### Terminal differentiation of porcine VM progenitors into mDA neurons

**Fig. 3:**
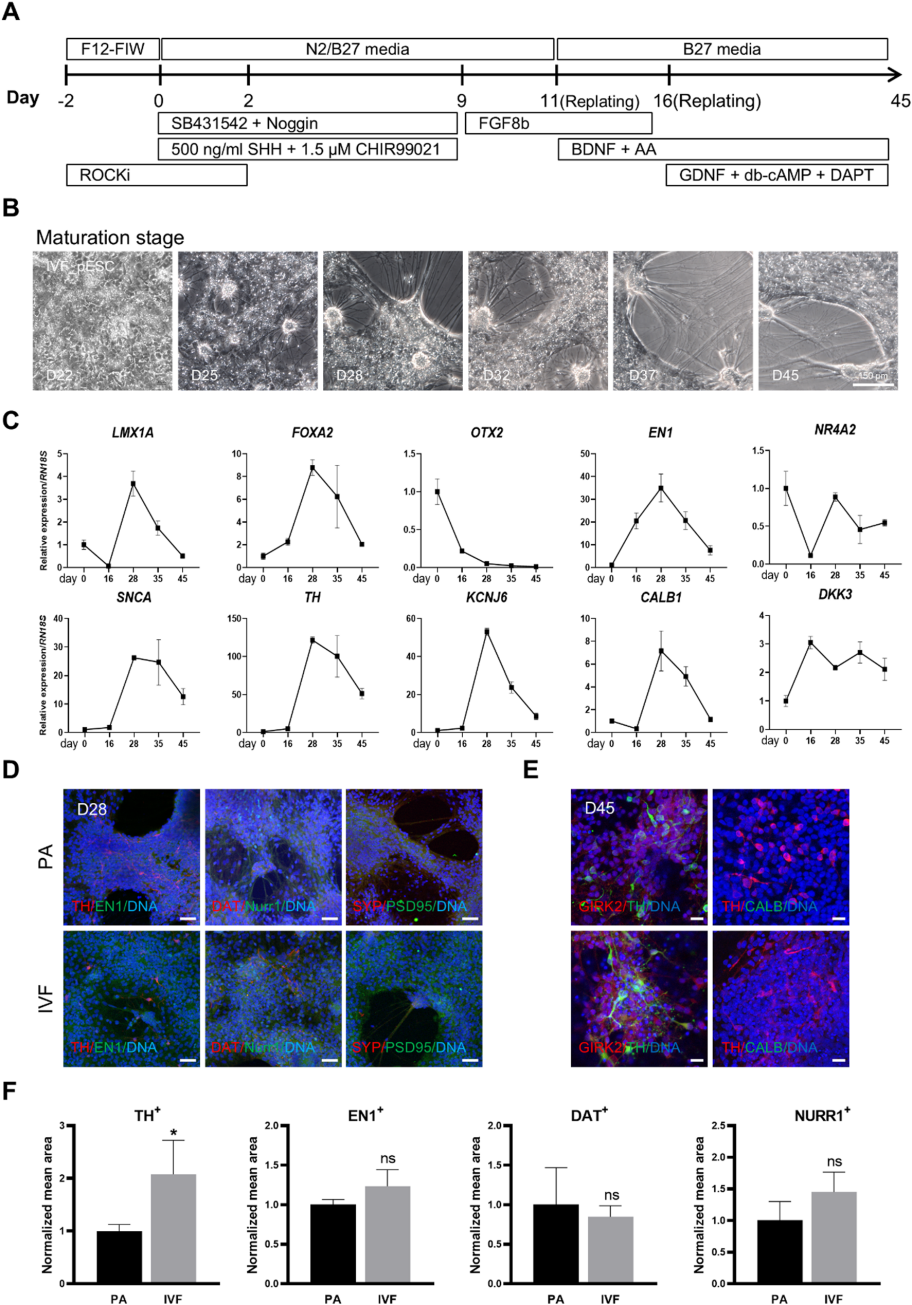
Terminal differentiation of porcine VM progenitors. (A) Schematic overview of the terminal differentiation protocol for midbrain dopaminergic (mDA) neurons with growth factors, small molecules, and culture medium. (B) Morphological change of IVF-pESC at different differentiation stages. (C) RT-qPCR analysis during terminal differentiation. (D) Immunostaining of TH/EN1, DAT/NURR1, and SYP/PSD95 at day 28 of differentiation. (E) Immunostaining of GIRK2/TH and TH/CALB at day 45 of differentiation. Scale bar, 20 μm. (F) Relative intensity of TH, EN1, DAT, and Nurr1 expression at day 28 of differentiation. ns, not significant; * *p* < 0.05

We investigated whether VM progenitors generated in vitro could differentiate into mDA neurons. Two lines of pESCs derived respectively from parthenogenetic activation (PA) and in vitro fertilization (IVF) were induced for terminal differentiation (Fig. 3A). By day 25 of differentiation, we observed neural dendrites in both cell lines (Figs. 3B and S3A). qRT-PCR analysis confirmed robust expression of mDA neuron-specific genes *LMX1A*, *FOXA2*, *EN1*, *SNCA*, *TH*, *KCNJ6*, and *CALB1* in differentiated PA and IVF cells on day 28 (Figs. 3C and S3B). Marker expression analysis at this timepoint confirmed the presence of mDA neuron markers TH, EN1, DAT, and Nurr1 in both PA and IVF cells (Figs. 3D). Notably, IVF cells exhibited a higher yield of TH-positive cells compared to PA cells (Fig. 3F). Additionally, synaptic proteins SYP and PSD95 were detected in both cell lines (Fig. 3D). By day 45 of differentiation, the A9 type mDA neuron marker GIRK2 was observed in both cell lines, while the A10 type marker CALB was absent (Fig. 3E). These results indicate that pESC-derived VM progenitors undergo in vitro maturation over an additional 12 days, exhibiting characteristics of A9 type mDA neurons.

### Functionality of porcine mDA neurons

**Fig. 4:**
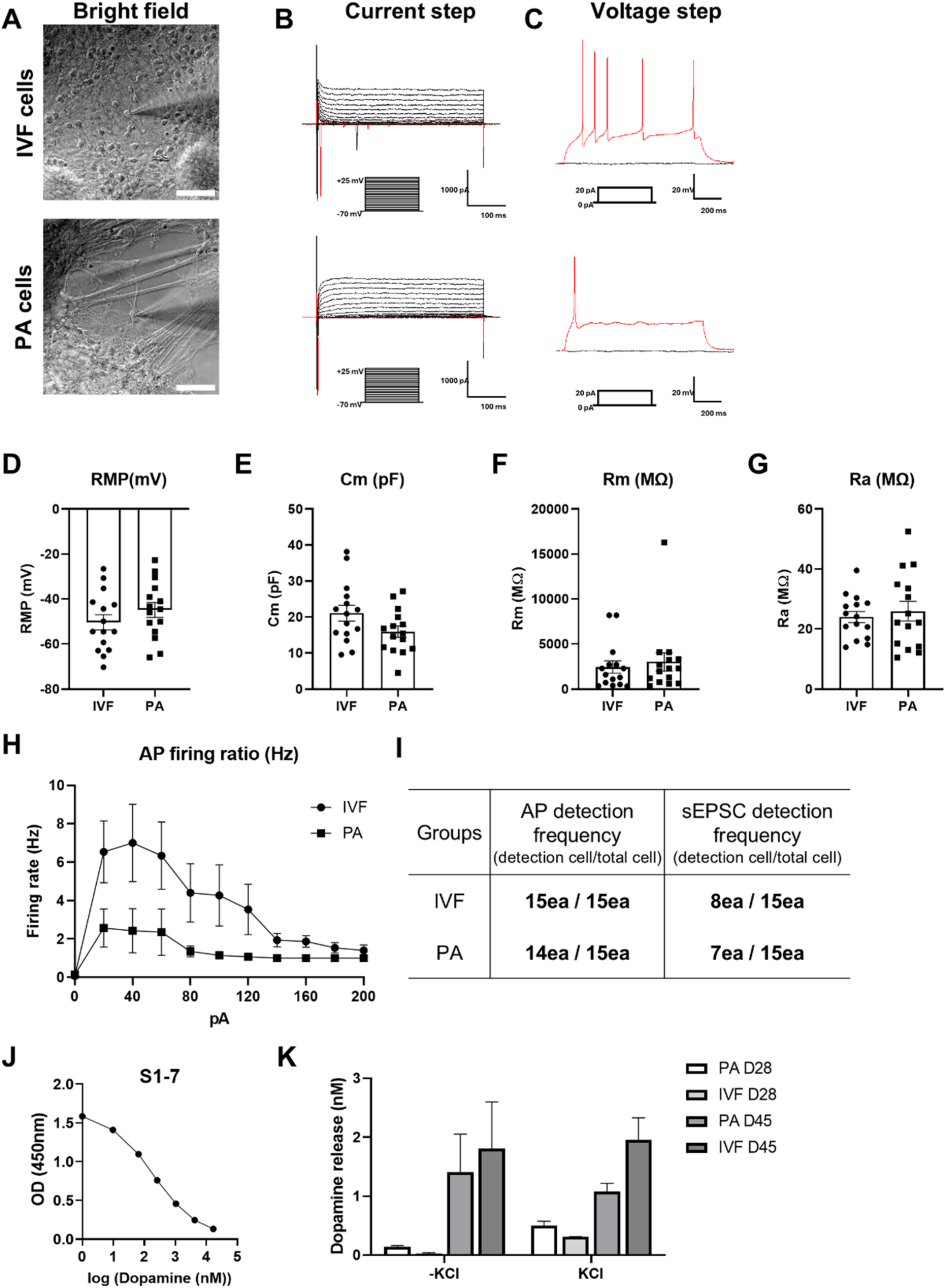
Functionality of the pESCs-derived mDA neurons. (A) Representative image of DA neurons from porcine IVF-ESCs or PA-ESCs that have undergone a patch clamp procedure. Scale bar, 65 μm. (B) Representative traces show the presence of voltage-dependent Na + and K + currents in DA neurons from porcine IVF-ESCs (upper) or PA-ESCs (bottom). (C) Representative trace of action potentials (AP) induced with depolarizing current injections in DA neurons from porcine IVF-ESCs (upper) or PA-ESCs (bottom). (D-G) Scatter plots with bars show analyses of the (D) Resting membrane potential (RMP), (E) Membrane capacitance (Cm), (F) Membrane resistance (Rm), and (G) Access resistance (Ra). (H) Comparison of responses (number of APs evoked by a 1 s stimulus) for the pESCs-derived DA neurons in different groups across a range of step current injections from 0 to 200 pA. I AP detection frequency and spontaneous excitatory post-synaptic currents (sEPSC) detection frequency of the porcine ESCs-derived DA neurons. (J) The standard curve for the dopamine release assay was created using standard samples of dopamine at specified concentrations. (K) On days 28 and 45, cultures of DA neurons derived from PA-ESCs or IVF-ESCs were stimulated with HBSS or KCl. Dopamine release was quantified using a competitive ELISA (*n* = 2)

We conducted electrophysiological recordings on day 30 differentiated cells (PA and IVF) to examine their functional properties (Fig. 4A). We confirmed the presence of ion channels in the cell membrane by measuring current steps at various voltages (Fig. 4B). Both PA and IVF cells displayed rebound APs following brief depolarization, a hallmark of mDA neurons, with PA cells showing wider APs compared to IVF cells (Fig. 4C and Table S3). The AP detection frequency was 100% in IVF cells and 93.3% in PA cells (Fig. 4I), indicating that nearly all differentiated cells had matured into neurons. The average RMP in both cell types, which is indicative of mature neuron properties, was less than − 40 mV (Fig. 4D and Table S3). The Cm, which correlates with cell surface area, was lower than previously reported values greater than ​​50 pF for hPSC-derived mDA neurons [39] and was instead ​​comparable to mouse values [40] (IVF: 21.1 ± 2.2 pF; PA: 15.9 ± 1.6 pF) (Fig. 4E and Table S3). In contrast, the Rm was similar to that of human mDA neurons [39] (IVF: 2457 ± 663; PA: 3010 ± 1002). The Ra, which reflects the sum of electrode resistance and resistance at the electrode-cell junction, was within the normal range for both cell types (IVF: 24; PA: 25.9). Data on AP firing ratios indicated that PA cells tended to generate a single APs, while IVF cells exhibited a multiple AP (4–6 Hz) pattern, characteristic of dopamine neurons in both mouse [41, 42] and human [38, 39, 43]. Lastly, we confirmed the presence of synaptic connectivity by observing the frequency of sEPSC in patched IVF (*n* = 8/15) and PA (*n* = 7/15) cells (Figs. 4I, S4A and B, and Table S3). In summary, the differentiated cells at day 30 from IVF and PA-pESCs exhibited mature electrophysiological properties, including high AP detection rates, low RMPs, low Rm values, and synaptic connectivity. However, IVF cells more closely resembled the characteristics of mDA neurons compared to PA cells, as indicated by a higher AP firing ratio. Dopamine release assays revealed a significant increase in basal dopamine secretion in both PA- and IVF-derived mDA neurons at day 45 compared to day 28 (Figs. 4J and K). However, KCl-induced stimulation elicited a more pronounced dopamine release response at day 28, suggesting greater excitability at an earlier stage of differentiation. These findings indicate a maturation-dependent shift in dopamine release dynamics, highlighting functional differences across developmental time points.

### Transcriptional capture of differentiated cells

**Fig. 5:**
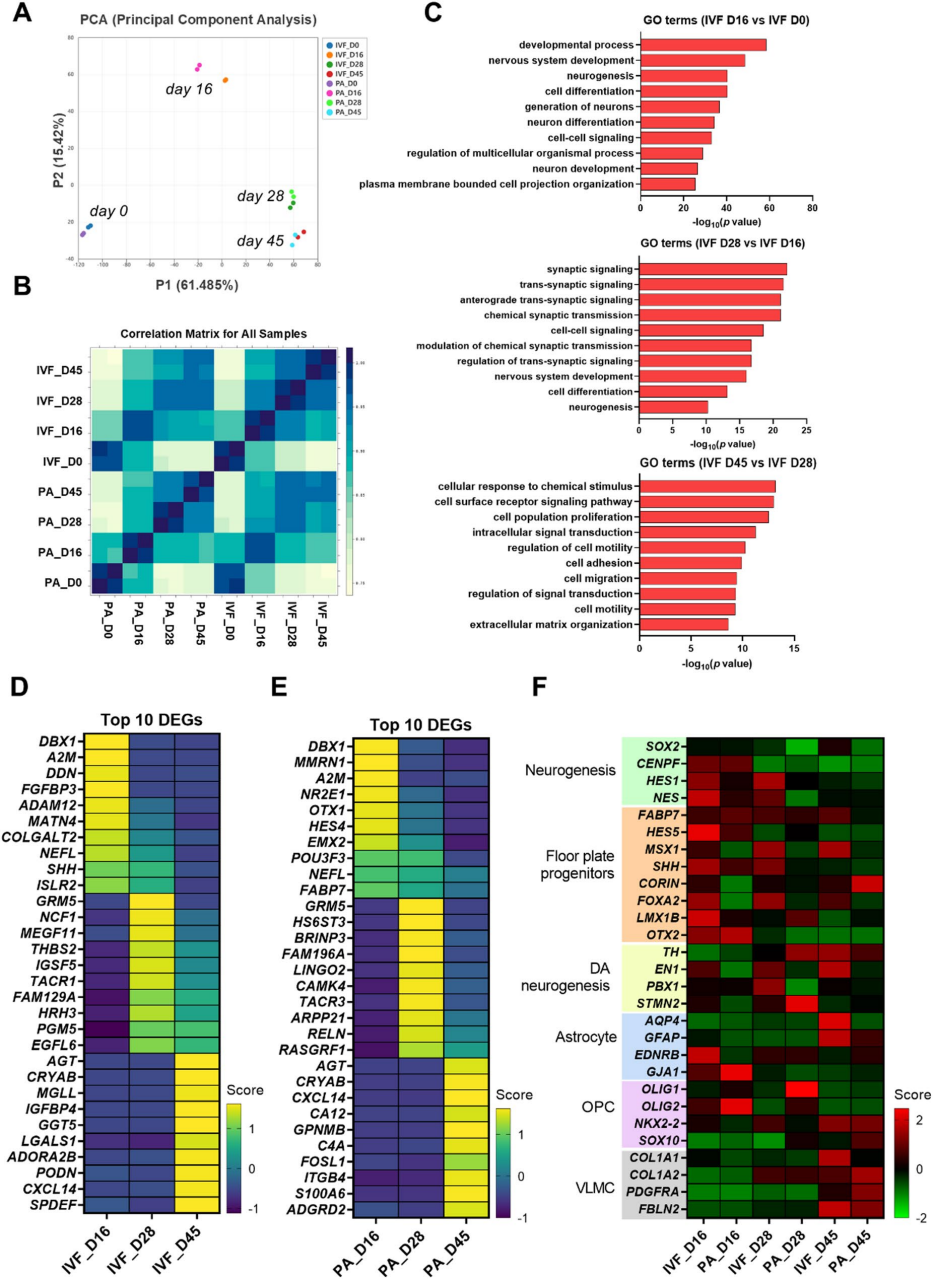
Transcriptome analysis of pESC-derived cells at different stages. (A) Principal Component Analysis (PCA) of the transcriptomic profiles of pESC-derived cells. (B) Sample distance plot for RNA expression among different time points of mDA differentiation. (C) Gene ontology (GO) enrichment analysis of differentially expressed genes (DEGs) in IVF cells between D0 and D16, D16 and D28, and D28 and D45 samples. (D and E) Top 10 DEGs across stages of differentiation in IVF (D) and PA (E) cells, respectively. (F) Heatmap of selected gene sets showing expression levels of established markers for neural progenitors, floor-plate progenitors, DA neurons, astrocytes, oligodendrocyte progenitors (OPCs), and vascular leptomeningeal cells (VLMC)

To investigate gene expression dynamics during the differentiation of mDA neurons, we conducted Bulk-RNA sequencing. Correlation analysis revealed strong clustering of samples by differentiation stage, regardless of their cell line origin (IVF or PA) (Fig. 5A). Notably, the correlation between samples from day 28 and day 45 was significantly higher than at other stages (Fig. 5B), suggesting potential transcriptomic similarities between these two time points. Gene Ontology (GO) analysis was performed to assess functional annotations at different time points. Both cell lines exhibited consistent patterns, with day 16 cells exhibiting upregulation of gene subsets related to neuronal processes, including neurogenesis, generation of neurons, and neuron development, compared to day 0 (Figs. 5C and S5). By day 28, there was an upregulation of gene subsets involved in synaptic functions, such as synaptic signaling, trans-synaptic signaling, and chemical synaptic transmission, relative to day 16. At day 45, gene expression shifted towards greater regulation of processes related to cellular response, including cellular response to chemical stimuli, cell motility, and cell migration, compared to day 28. The top 10 DEGs across various stages of differentiation highlighted specific genes associated with neural development on day 16, neurotransmitter signaling and synaptic formation on day 28, and cellular stress response and immune modulation on day 45 (Figs. 5D and E). When comparing the expression levels of gene sets associated with mDA neuron development, IVF cells exhibited higher expression regardless of differentiation time compared to PA cells (Fig. 5F). Notably, in differentiated cells at day 45, there was a significant expression of genes related to various cell types, including astrocytes, oligodendrocyte progenitors (OPCs), and vascular leptomeningeal cells (VLMCs), which are present in the adult midbrain [44]. These findings indicate a temporal progression in gene expression during mDA neuron differentiation, with early stages focused on neuronal development, mid-stages on synapse formation, and later stages on cellular responses, highlighting the dynamic nature of neuronal maturation and function over time.

### Generation of porcine midbrain-like organoids

**Fig. 6:**
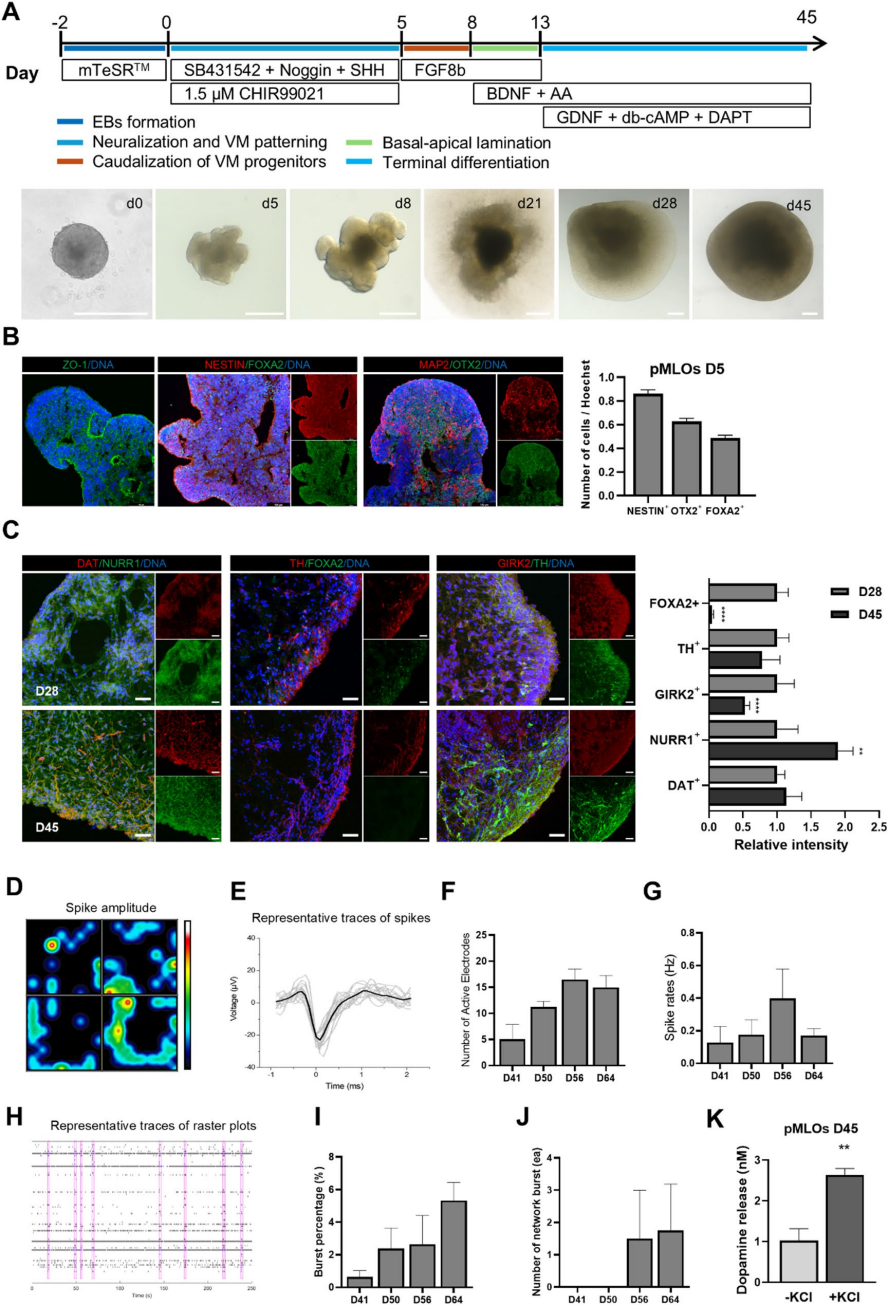
Generation and characterization of porcine midbrain-like organoids (pMLOs) from IVF-pESCs. (A) Schematic representation of the differentiation protocol used to generate pMLOs from IVF-pESCs. The timeline indicates the addition of signaling molecules at specific time points. Representative bright-field images show morphological changes from embryoid body (EB) formation (Day 0) to organoid maturation (Day 45). Scale bars, 500 μm. (B) Immunofluorescence analysis of pMLOs at Day 5. OTX2 and FOXA2 indicate midbrain identity, while ZO-1, NESTIN, and MAP2 mark structural organization. The bar graph quantifies the proportion of NESTIN+, OTX2+, and FOXA2 + cells. Scale bars, 20 μm. (C) Immunostaining of pMLOs at Days 28 and 45. DAT, NURR1, and TH are detected in both time points, while FOXA2 expression is observed at Day 28. The bar graph quantifies the relative intensity of marker expression between the two time points. Scale bars, 20 μm. (D-G) Multi-electrode array (MEA) recordings of pMLOs. (D) Heatmap representing spike amplitude distribution. (E) Representative traces of recorded spikes. (F-J) Quantification of neural activity over time, including (F) number of active electrodes, (G) spike rate, (I) burst percentage, and (J) number of network bursts. (K) Dopamine release assay of pMLOs at Day 45. The bar graph shows dopamine release levels under basal and KCl-stimulated conditions. *P* < 0.01, Student’s t-test

Given that IVF-derived pESCs exhibited superior dopaminergic marker expression and functional maturation compared to PA-derived cells under 2D conditions, only the IVF-derived line was utilized for organoid generation. The established protocol for inducing mDA neurons was adapted to a three-dimensional (3D) culture system to generate porcine midbrain-like organoids (pMLOs) (Fig. 6A). Exposure to SHH and intermediate concentrations of CH promoted the formation of neuroepithelium-like buds (marked by ZO-1, NESTIN, and MAP2) with floor plate characteristics (indicated by OTX2 and FOXA2) by day 5 of EB formation (Fig. 6B). By day 28, mature DA neurons expressing DAT, NURR1, and TH were identified, while by day 45, FOXA2 expression was lost, and NURR1 expression significantly increased (Fig. 6C). The functionalities of pMLOs were assessed through MEA analysis, which demonstrated stable attachment and an increase in spike amplitude over time (Figs. 6D and E). Quantitative analysis revealed a progressive rise in active electrodes (Fig. 6F), spike rates (Fig. 6G), burst percentage (Fig. 6I), and network bursts (Fig. 6J), indicating enhanced neuronal activity. Notably, dopamine release assays showed a significantly higher KCl-induced response in pMLOs (Fig. 6K) compared to monolayer conditions at the same differentiation stage (Fig. 4K), highlighting the enhanced functional maturation in a 3D environment.

### Single-cell RNA sequencing reveals dynamic transcriptional transitions in pMLOs

**Fig. 7:**
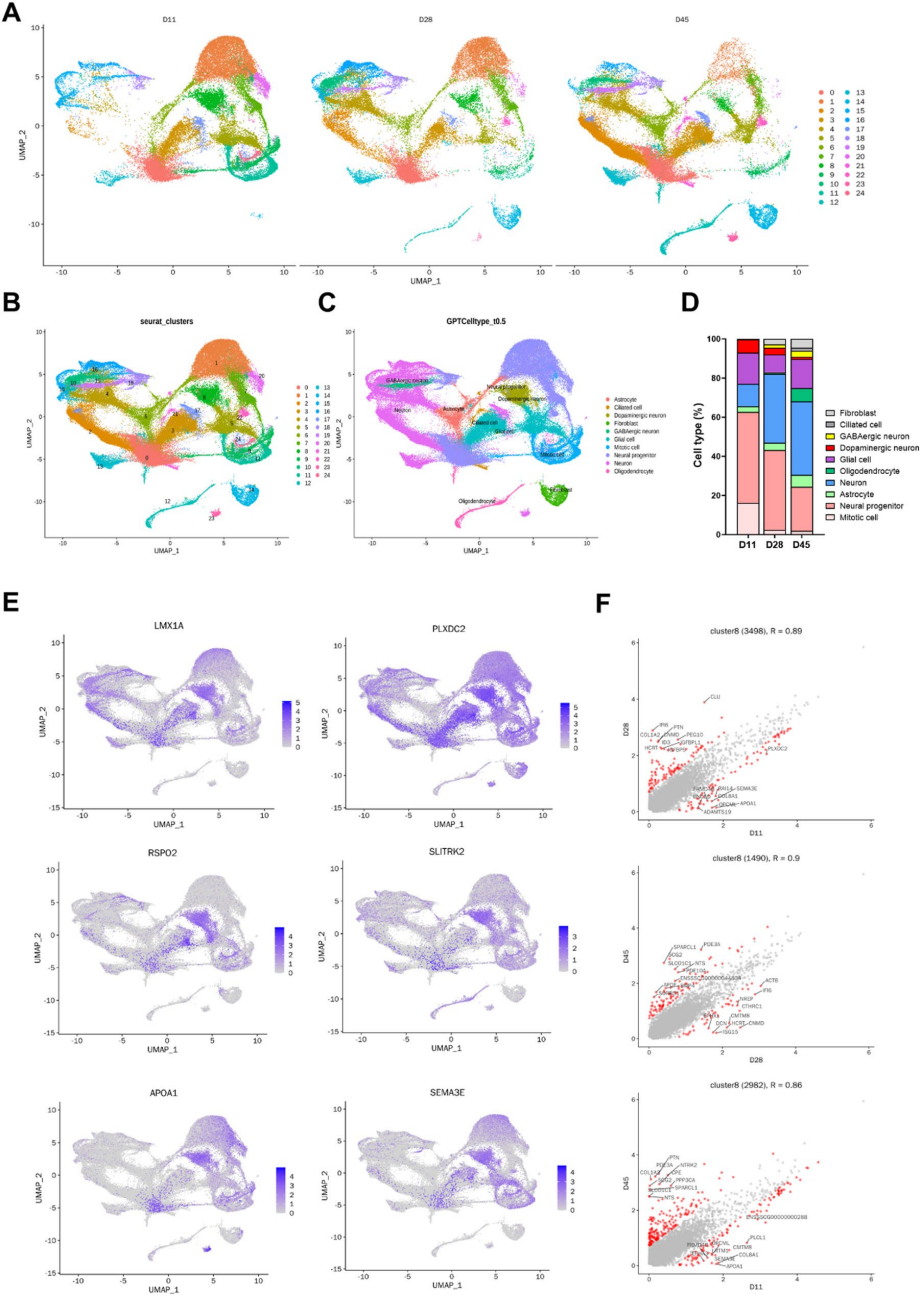
Single-cell transcriptomic profiling of pMLOs across differentiation stages. (A) UMAP plots of single-cell RNA-seq data from pMLOs at days 11, 28, and 45, generated from biological duplicates, showing distinct clustering patterns across differentiation stages. (B) UMAP visualization of integrated datasets from all-time points, revealing discrete cell clusters. (C) Cell type annotation based on canonical marker gene expression, identifying major neural and glial populations. (D) Proportional distribution of each cell type across differentiation stages. (E) Feature plots showing the expression of dopaminergic neuron–associated genes (*LMX1A*, *PLXDC2*, *RSPO2*, *SLITRK2*, *APOA1*, *SEMA3A*) across all time points on the UMAP embedding. (F) Scatter plots showing differentially expressed genes in cluster 8 (dopaminergic neurons) between D11 vs. D28, D28 vs. D45, and D11 vs. D45

Single-cell RNA sequencing was performed on pMLOs at days 11, 28, and 45 of differentiation to characterize temporal changes in cellular composition and transcriptional dynamics. After alignment to the *Sus scrofa* Sscrofa11.1 genome and downstream processing, 103,310 high-quality cells were obtained and visualized via UMAP, revealing time-dependent shifts in clustering patterns (Fig. 7A). Integration across all time points identified discrete cell clusters (Fig. 7B), which were annotated based on canonical marker expression (Fig. 7C). Cell type proportions varied by stage, with progenitor-like populations enriched at D11 and neuronal and glial cell types, including oligodendrocytes and GABAergic neurons, increasingly represented at later stages (Fig. 7D). A dopaminergic neuron cluster emerged as early as D11, characterized by expression of markers including *LMX1A*, *RSPO2*, *APOA1*, *PLXDC2*, *SLITRK2*, and *SEMA3A* (Figs. 7E and S6A), which have been implicated in lineage specification, axon guidance, synaptic organization, and neuroprotection in dopaminergic neurons [45–49]. Cluster-specific expression analysis revealed that *PLXDC2*, *CLU*, and *PDE3A* were most highly expressed in dopaminergic neurons (cluster 8) at D11, D28, and D45, respectively, suggesting temporal regulation of genes associated with axon targeting, synaptic stabilization, and intracellular signaling. Although the proportion of dopaminergic neurons was modest, these cells exhibited strong expression of canonical dopaminergic markers such as *TH*, *NR4A2*, *KCNC2*, and *SCN2A*, while forebrain (*FOXG1*) and hindbrain (*HOXA2*) markers were largely absent (Figs. 7F and S6B), supporting the midbrain identity of these neurons and the regional specificity of the differentiation protocol. Together, these findings highlight the emergence, molecular identity, and progressive maturation of dopaminergic neurons within pMLOs.

## Discussion

The present study attempted for the first time to induce caudal VM patterning in porcine stem cells using GSK3i, SHH, and FGF8, and then mature them into mDA neurons in vitro. Concentrations of CH, a GSK3i, play a crucial role in determining the developmental fate of the forebrain, midbrain, and hindbrain during neural induction [29]. In humans, the absence of GSK3i leads cells to adopt a forebrain fate [50], while concentrations of 0.6–1.0 µM direct them towards a midbrain fate, and concentrations of 1.5–4.0 µM promote a hindbrain fate [51]. By examining the expression of the caudal VM markers (Fig. 1), the optimal concentration of GSK3i required to induce porcine midbrain fate was determined to be 1.5 µM, which is higher than the concentration used in humans. The reason for differences in required concentration of GSK3i might be species-specific differences in WNT signaling pathways and developmental stage differences.

During development, the floor plate is located along the ventral midline of the vertebrate neural tube and serves as the origin of mDA neurons [52, 53]. In the midbrain, SHH and FOXA2 collaborate to define the identity of VM progenitors [54]. In this study, it was shown that inducing porcine VM progenitors with 300 ng/ml SHH, a concentration used in human stem cells [29, 38], resulted in a lack of expression of the floor plate marker FOXA2 (Fig. 2). An increase in FOXA2 expression and a decrease in the level of PAX6 were observed when the 500 ng/ml SHH was supplemented to the induction media. These findings suggest that porcine cells may be less sensitive to SHH signaling in establishing the VM region, or that a more complex regulatory mechanism requiring higher SHH signaling is involved.

In this study, PA and IVF embryo-derived pESCs were used to induce differentiation into mDA neurons. By day 28 of differentiation, both cell lines exhibited the expression of relevant markers and electrophysiological activities, including AP and synaptic connections (Figs. 3 and 4). These results indicate that the maturation period of mDA neurons in porcine is shorter than the 45 + days observed in humans [38, 39, 43]. This difference is linked to the varying durations of brain development between the two species (20 years and 6 months in humans and pigs, respectively) [55]. Notably, the IVF-derived cells demonstrated a higher yield of TH-positive cells (Fig. 3F), and a greater AP firing ratio (Fig. 4H) compared to the PA cells. This suggests that IVF-derived pESCs are more effective at generating functional mDA neurons than their PA-derived counterparts. This advantage arises from inherent differences in epigenetic or genetic stability, which could enhance their responsiveness to differentiation signals. Supporting this, a recent study using single-cell RNA sequencing of porcine embryos reported distinct transcriptomic and epigenetic profiles between IVF and PA embryos, including differential dynamics in zygotic genome activation, maternal RNA decay, and the expression of chromatin-modifying enzymes such as DNMT1, DNMT3A/B, and UHRF1/2 [56]. These molecular differences may underlie the improved lineage commitment observed in IVF-derived pESCs. Further analysis of these factors may elucidate the mechanisms by which embryonic origin affects stem cell differentiation, potentially improving protocols for generating clinically relevant mDA neurons.

The results of the bulk RNA sequencing provide porcine-specific dynamic changes in gene expression that occur during the differentiation of mDA neurons in both IVF and PA cells (Fig. 5). The stepwise gene expression profiles identified in the porcine model reveal key regulatory factors involved in the functional maturation of mDA neurons. Notably, the relatively high expression of VLMC-related genes in 45-day differentiated cells (Fig. 5F) suggests the emergence of supportive stromal cell types, reflecting a maturing midbrain-like niche conducive to functional integration [57, 58]. This insight provides an opportunity to investigate the relationship between these factors and specific pathological changes by utilizing porcine cells at various stages of differentiation in neurological disease modeling. Furthermore, the differences in gene expression between IVF and PA cells offer valuable insights for assessing the impact of genetic and epigenetic factors on the efficiency of neuronal differentiation. This understanding can lead to the development of optimized differentiation protocols, enhancing their clinical applicability.

The adaptation of differentiation media for inducing mDA neurons in a 3D environment has successfully produced midbrain organoids. During neural induction, an expanded neuroepithelium structure formed within the organoids by day 5 (Fig. 6B). These characteristics are different from the multiple and independent neuroepithelium units that appear during development of human VM organoids [59–61]. In contrast, these expanded structures have been shown to form in human cortical organoids through a gradient of TGF-β signaling [62]. Therefore, the expanded epithelium structures within porcine midbrain organoids are likely induced by a gradient of TGF-β1 contained in the mTeSR medium during the medium switch process. These structures enhance the homogeneity of the organoids and reflect a single neural tube, resembling in vivo conditions. However, despite this morphogenetic advantage, pMLOs appeared to exhibit less distinct layered organization and a lower proportion of cells expressing mature dopaminergic neuron markers compared to human VM organoids, suggesting potential limitations in achieving full neuronal subtype specification. In addition to the morphogenetic features observed during early induction, further characterization of pMLOs revealed progressive molecular and functional maturation of dopaminergic neurons over time. The sequential expression of key markers including NESTIN, OTX2, and FOXA2 at early stages, and TH, DAT, and NURR1 at later stages (Fig. 6B and C) suggest that the temporal patterning of differentiation in pMLOs closely mimics midbrain DA neuron development in vivo. Notably, FOXA2 expression diminished by day 45, coinciding with elevated NURR1 levels (Fig. 6C), indicating a shift from progenitor to mature neuronal identity. Interestingly, Single-cell RNA sequencing further revealed that several canonical dopaminergic neuron markers—such as *TH*, *NR4A2*, *KCNC2*, and *SCN2A*—were not exclusively confined to the dopaminergic neuron cluster but were also expressed in other neuronal clusters, with their expression levels progressively increasing over differentiation time points (Figure S6B). This distribution may reflect the gradual acquisition of dopaminergic identity across a spectrum of transcriptional states, a phenomenon previously reported in single-cell analyses of midbrain development in mouse and human models [63, 64]. For example, *NR4A2* plays a crucial role not only in the early specification of dopaminergic precursors but also in the maintenance of mature dopaminergic neurons [65]. Moreover, *KCNC2* and *SCN2A*, while enriched in dopaminergic neurons, are associated with general neuronal excitability and may be expressed in a broader set of maturing neurons during in vitro differentiation [66]. The scRNA-seq data thus suggest that the expression of dopaminergic markers may emerge in a more distributed and temporally dynamic manner than inferred from bulk or static analyses. This may be due to transcriptional plasticity and asynchronous lineage progression commonly observed in organoid systems [67, 68], where spatial constraints and morphogen gradients are less tightly regulated than in vivo. Such observations emphasize the importance of using high-resolution single-cell approaches to capture the full spectrum of dopaminergic neurogenesis in large-animal models like the pig. Collectively, these results highlight the utility of pMLOs as a physiologically relevant in vitro model for studying midbrain development and dopaminergic neuron function in a large-animal system.

Previous studies have reported the induction of DA-like cells from porcine pluripotent stem cells; however, most relied solely on the expression of TH without further molecular or functional validation. For instance, Hwang et al. [69] demonstrated neural differentiation in 3D organoids but assessed only TH and MAP2, while Yang et al. [70] identified TH-positive grafts following transplantation without in-depth characterization. In contrast, our study evaluated additional DA-specific markers such as DAT, confirmed functionality through electrophysiological and transcriptomic analyses, and recapitulated DA neuron development under both 2D and 3D conditions. Although in vivo grafting was not performed, this study represents, to our knowledge, the most comprehensive in vitro characterization of functional porcine DA neurons reported to date.

Taken together, our findings provide critical insight into the selection of optimal strategies for generating dopaminergic neurons in large-animal models. While both IVF- and PA-derived pESCs demonstrated competence in mDA neuron differentiation, the consistently higher yield and functional maturation observed in IVF-derived cells suggest that they are more suitable for translational applications. Furthermore, the ability of IVF-pESCs to generate both monolayer-derived neurons and self-organizing midbrain-like organoids enhances their versatility. For disease modeling and high-throughput drug screening, organoid-based systems may offer a more physiologically relevant platform due to their 3D architecture and cellular diversity. Conversely, monolayer cultures derived from IVF-pESCs may be more appropriate for cell-based therapies requiring scalable production and precise quality control. Therefore, the combination of IVF-pESCs with application-specific culture formats represents a preferred method for the future development of porcine DA neuron platforms across diverse biomedical applications.

## Conclusions

This study establishes an efficient protocol for deriving functional mDA neurons and pMLOs from pESCs. We demonstrate that porcine cells require higher levels of GSK3 and SHH signaling to achieve ventral midbrain identity compared to human protocols. IVF-derived pESCs showed superior dopaminergic differentiation and maturation compared to PA-derived cells. pMLOs formed early neuroepithelial structures and exhibited synchronized neuronal activity and dopamine release. Single-cell transcriptomics revealed the progressive and distributed acquisition of dopaminergic identity. These findings highlight the value of pMLOs as a large-animal in vitro model for studying neurodevelopment and disease, offering a scalable and translationally relevant alternative to in vivo pig models.

## Supplementary Information

Below is the link to the electronic supplementary material.

Supplementary Material 1.

## Data Availability

The bulk RNA sequencing data generated in this study have been deposited in the Gene Expression Omnibus (GEO) under accession number GSE296088, and the single-cell RNA sequencing data are available under accession number GSE296089.

## Abbreviations

3D: Three-dimensional
APs: Action potential(s)
cDNA: Complementary DNA
CH: CHIR99021
Cm: Membrane capacitance
DEGs: Differentially expressed genes
GO: Gene Ontology
IVF: In vitro fertilization
LN111: Laminin-111
MEA: Microelectrode array
NHPs: Non-human primates
OPCs: Oligodendrocyte progenitors
PA: Parthenogenetic activation
PCA: Principal component analysis
PD: Parkinson’s disease
pESCs: Porcine embryonic stem cells
pMLOs: Porcine midbrain-like organoids
Ra: Access resistance
Rm: Membrane resistance
RMP: Resting membrane potential
scRNA-seq: Single-cell RNA sequencing
SEM: Standard error of the mean
sEPSC: Spontaneous excitatory postsynaptic currents
SN: Substantia nigra
UMAP: Uniform Manifold Approximation and Projection
VLMCs: Vascular leptomeningeal cells
VM: Ventral midbrain
