# Supplementary material for: In vitro derivation of midbrain dopaminergic neurons from porcine embryonic stem cells in multi-dimensional conditions
Source: Stem Cell Res Ther. 2025 Nov 5;16:617. doi: 10.1186/s13287-025-04693-9 (PMC12590826; doi:10.1186/s13287-025-04693-9)
Supplement: Supplementary file 1 — Supplementary Material 1. [file 13287_2025_4693_MOESM1_ESM.docx]

**Supplementary Information**

***In vitro* Derivation of Midbrain Dopaminergic Neurons from Porcine Embryonic Stem Cells in Multi-dimensional Conditions**

Hyerin Choi^1,2,5^, Dongjin Oh^1,2,5^, Ali Jawad^1,2^, Zheng Haomiao^1,2^, Jaehyung Ham^1,2^, Juyoung Heo^1,2^, Aram Oh^1,2^, Huijin Jin^1,2^, Jaehyeok Seo^1,2^, and Sang-Hwan Hyun^1,2,3,4*^

^1^Veterinary Medical Center and College of Veterinary Medicine, Laboratory of Veterinary Embryology and Biotechnology (VETEMBIO), Chungbuk National University, Cheongju, Republic of Korea,

^2^Institute of Stem Cell and Regenerative Medicine (ISCRM), Chungbuk National University, Cheongju, Republic of Korea,

^3^Vet-ICT Convergence Education and Research Center (VICERC), Chungbuk National University, Cheongju, Republic of Korea,

^4^Chungbuk National University Hospital, Cheongju, Republic of Korea.

^5^Department of Neurology, Institute for Cell Engineering, School of Medicine, Johns Hopkins Medicine, Baltimore, ML, USA

***Correspondence**: Professor Sang-Hwan Hyun, DVM, Ph.D

Laboratory of Veterinary Embryology and Biotechnology (VETEMBIO)

College of Veterinary Medicine, Chungbuk National University

1 Chungdae-ro, Seowon-gu, Cheongju 28644, Republic of Korea

Tel: +82-43-261-3393; Fax: +82-43-267-3150; E-mail: [shhyun@cbu.ac.kr](mailto:shhyun@cbu.ac.kr)

**Supplemental Tables**

**Table S1. Antibodies list for immunofluorscence**

| **Antibodies** | **Cat. No.** | **Company** |
| --- | --- | --- |
| Mouse HNF-3 beta/FoxA2 Antibody (OTI3C10) | NBP2-02088 | Novus Biologicals |
| Mouse OTX2 Monoclonal Antibody (1H12C4B5) | MA5-15854 | Thermo Fisher Scientific |
| Mouse EN1 Antibody | 4G11 | Developmental Studies Hybridoma Bank |
| Rabbit BARHL1 Polyclonal Antibody | NBP1-86513 | Novus Biologicals |
| Rabbit Tyrosine Hydroxylase Monoclonal Antibody (E2L6M) | 58844S | Cell Signaling Technology |
| Mouse Tyrosine Hydroxylase Monoclonal Antibody (LNC1) | 45648S | Cell Signaling Technology |
| Rabbit MAP2 Antibody | 4542S | Cell Signaling Technology |
| Mouse GFAP Monoclonal Antibody (GA5) | 3670 | Cell Signaling Technology |
| Mouse Pax-6 (PAX6) Monoclonal Antibody | sc-81649 | Santa Cruz Biotechnology |
| Rabbit Dopamine Transporter Polyclonal Antibody | PA5-78382 | Thermo Fisher Scientific |
| Mouse Nurr1 Monoclonal Antibody (N1404) | MA1195 | Thermo Fisher Scientific |
| Rabbit Synaptophysin Monoclonal Antibody (SP11) | MA514532 | Thermo Fisher Scientific |
| Mouse PSD-95 Monoclonal Antibody (6G6-1C9) | MA1045 | Thermo Fisher Scientific |
| Rabbit Anti-GIRK2 (Kir 3.2) Antibody | APC-006 | Alomone Labs |
| Mouse Calbindin D28K antibody (D-4) | Sc-365360 | Santa Cruz Biotechnology |
| Mouse ZO-1 Monoclonal Antibody (ZO1-1A12) | 33-9100 | Thermo Fisher Scientific |
| Rabbit Anti-Nestin Antibody, clone 10C2 | MAB5326 | Thermo Fisher Scientific |
| Rabbit β3-Tubulin (D71G9) XP Monoclonal Antibody | 5568 | Cell Signaling Technology |
| Mouse BD Pharmingen™ Purified Anti-Ki-67 | 550609 | BD Biosciences |

**Table S2. Primer sequences for qRT-PCR**

| **mRNA** | **Primer sequences** | | **GenBank**  **accession number** |
| --- | --- | --- | --- |
| *RN18S* | F: 5'-CGCGGTTCTATTTTGTTGGT-3' | R: 5'-AGTCGGCATCGTTTATGGTC-3' | NR_046261.1 |
| *EN1* | F: 5'-TGCTAAGTACCCAGACCACA-3' | R: 5'-CTTCTTCAGCTTCCTCGTTCG-3' | XM_003133282.4 |
| *SPRY1* | F: 5'-CAAATCAAGGCCGTCAGAGG-3' | R: 5'-GCTGGTAGGTCTATGCTCGT-3' | NM_001267835.1 |
| *CNPY1* | F: 5'-CCAGGAGGGGAGACGAAATA-3' | R: 5'-CTTGTCAGCGAGGTGGTTTG-3' | XM_021078820.1 |
| *ETV5* | F: 5'-GGGGAGCGATACGTCTACAA-3' | R: 5'-GGAGGTAAGCAGGGTTGTCT-3' | XM_021068467.1 |
| *FOXG1* | F: 5'-CCCTACTACCGCGAGAACAA-3' | R: 5'-GTGCCGCCTATGAACACATC-3' | XM_021099188.1 |
| *NKX2.1* | F: 5'-CAAGAATATGGCCCCGCTAC-3' | R: 5'-GTGTCAGGTGGATCATGCTG-3' | XM_005666203.3 |
| *HOXA2* | F: 5'-GGATGAAGCACAAGAGGCAG-3' | R: 5'-AGTCGCCATTGTGTCCATTG-3' | XM_003134843.5 |
| *GBX2* | F: 5'-GGTGCAGGTGAAAATCTGGT-3' | R: 5'-GCTGACTTCTAATGGCGAACC-3' | XM_003133755.5 |
| *PAX6* | F: 5'-CAGGGCAATCGGTGGTAGTA-3' | R: 5'-CCAGGTTGCGAAGAACTCTG-3' | XM_005661042.3 |
| *FOXA2* | F: 5'-CATCTCGCTCATCACCATGG-3' | R: 5'-AGGAAGCAGTCGTTGAAGGA-3' | XM_005672754.3 |
| *LMX1A* | F: 5'-GAGACCACATGCACCCCTAT-3' | R: 5'-AGTACAGGTGGTCAATGGGG-3' | XM_013988078.2 |
| *OTX2* | F: 5'-GGTCCTACCCCATGACCTAT-3' | R: 5'-GTACCCATGGGACTGAGTGT-3' | XM_021102521.1 |
| *NR4A2* | F: 5'-GCACAACTACCAGCAACACA-3' | R: 5'-GCGTTTTCCTCTGCTCGATC-3' | XM_021074549.1 |
| *SNCA* | F: 5'-AGCAGCGGGAAAGACAAAAG-3' | R: 5'-CATTCTTGCCCAGCTGATCC-3' | XM_021100514.1 |
| *TH* | F: 5'-AATTGAGAAGCTGTCCACGC-3' | R: 5'-TGGCGTCACTGAAACTCTCA-3' | XM_021085452.1 |
| *KCNJ6* | F: 5'-AAATCCAAAGGTACGTGCGG-3' | R: 5'-AGCCACCAGATCATCCCAAA-3' | XM_021070978.1 |
| *CALB1* | F: 5'-GAATCCCACCTGCAATCACC-3' | R: 5'-CCAATCCAGCCTTCTTTCGT-3' | NM_001130226.1 |
| *DKK3* | F: 5'-TGATGGAGCTTTGGACCGAT-3' | R: 5'-TGAAGCCTCCATCCTCGTAC-3' | XM_021082083.1 |

F: Forward, R: Reverse

**Table S3. Whole-Cell Patch Clamp Analysis of pESC-derived mDA neurons**

| Groups | No | RMP(mV) | Cm (pF) | Rm (MΩ) | Ra (MΩ) | Action potential firing ratio (Hz) | | | | | | | | | | | AP detection (% of total cell) | sEPSC detection (% of total cell) |
| --- | --- | --- | --- | --- | --- | --- | --- | --- | --- | --- | --- | --- | --- | --- | --- | --- | --- | --- |
|  |  |  |  |  |  | 0 | 20 | 40 | 60 | 80 | 100 | 120 | 140 | 160 | 180 | 200 |  |  |
| IVF | 1 | -63.0 | 25.72 | 2300 | 28.6 | 0 | 8 | 7 | 6 | 5 | 5 | 5 | 5 | 4 | 4 | 5 | positive | positive |
|  | 2 | -30.7 | 22.2 | 364.9 | 25.2 | 1 | 2 | 3 | 2 | 1 | 1 | 2 | 1 | 2 | 1 | 1 | positive | negative |
|  | 3 | -49.9 | 27.98 | 1300 | 24.1 | 0 | 10 | 16 | 19 | 21 | 22 | 21 | 5 | 4 | 4 | 2 | positive | negative |
|  | 4 | -59.3 | 18.18 | 1100 | 30.4 | 0 | 7 | 3 | 2 | 2 | 2 | 2 | 1 | 1 | 1 | 1 | positive | positive |
|  | 5 | -54.5 | 36.34 | 1600 | 21.7 | 0 | 14 | 17 | 5 | 3 | 3 | 2 | 2 | 2 | 1 | 2 | positive | positive |
|  | 6 | -35.3 | 13.42 | 4100 | 16.9 | 0 | 1 | 1 | 1 | 1 | 1 | 1 | 1 | 1 | 1 | 1 | positive | positive |
|  | 7 | -44.4 | 15.63 | 2700 | 39.5 | 0 | 19 | 22 | 14 | 3 | 2 | 2 | 2 | 1 | 1 | 1 | positive | positive |
|  | 8 | -26.6 | 20.88 | 723.9 | 20.8 | 0 | 4 | 5 | 4 | 3 | 3 | 2 | 2 | 2 | 2 | 1 | positive | negative |
|  | 9 | -54.6 | 10.13 | 8200 | 14.8 | 0 | 1 | 1 | 1 | 1 | 1 | 1 | 1 | 1 | 1 | 0 | positive | negative |
|  | 10 | -54.5 | 23.73 | 8200 | 13.9 | 0 | 16 | 21 | 21 | 2 | 2 | 2 | 2 | 2 | 1 | 1 | positive | positive |
|  | 11 | -42.6 | 15.35 | 2300 | 20.7 | 0 | 6 | 3 | 3 | 3 | 2 | 2 | 1 | 1 | 1 | 1 | positive | positive |
|  | 12 | -41.4 | 9.5 | 2700 | 15.9 | 0 | 10 | 2 | 2 | 2 | 2 | 2 | 1 | 1 | 1 | 1 | positive | positive |
|  | 13 | -65.5 | 38.13 | 409.4 | 27.5 | 0 | 0 | 0 | 2 | 2 | 1 | 1 | 1 | 1 | 1 | 1 | positive | negative |
|  | 14 | -62.7 | 22.02 | 332 | 28.1 | 0 | 0 | 0 | 1 | 1 | 1 | 1 | 1 | 1 | 1 | 1 | positive | negative |
|  | 15 | -70.2 | 16.68 | 533.3 | 31.7 | 0 | 0 | 4 | 12 | 16 | 16 | 7 | 3 | 4 | 2 | 2 | positive | negative |
|  | AV | -50.3 | 21.1 | 2457.6 | 24.0 | 0.1 | 6.5 | 7.0 | 6.3 | 4.4 | 4.3 | 3.5 | 1.9 | 1.9 | 1.5 | 1.4 | 15ea / 15ea | 8ea / 15ea |
|  | SD | 13.2 | 8.5 | 2570.9 | 7.2 | 0.3 | 6.2 | 7.8 | 6.8 | 5.9 | 6.2 | 5.1 | 1.4 | 1.2 | 1.1 | 1.1 | 100.0 | 53.3 |
|  | SE | 3.4 | 2.2 | 663.8 | 1.9 | 0.1 | 1.6 | 2.0 | 1.8 | 1.5 | 1.6 | 1.3 | 0.4 | 0.3 | 0.3 | 0.3 |  |  |
| PA | 1 | -50.6 | 17.92 | 16300 | 41.1 | 0 | 7 | 3 | 2 | 1 | 2 | 1 | 1 | 1 | 1 | 1 | positive | positive |
|  | 2 | -43.4 | 22.23 | 1200 | 25.2 | 0 | 2 | 2 | 1 | 1 | 1 | 1 | 1 | 1 | 1 | 1 | positive | positive |
|  | 3 | -27.8 | 25.68 | 1300 | 41.5 | 0 | 2 | 1 | 1 | 1 | 1 | 1 | 1 | 1 | 1 | 1 | positive | negative |
|  | 4 | -54.6 | 15.43 | 2700 | 34.9 | 1 | 2 | 1 | 1 | 1 | 1 | 1 | 1 | 1 | 1 | 1 | positive | positive |
|  | 5 | -66.0 | 27.16 | 2300 | 15.2 | 0 | 14 | 17 | 18 | 5 | 2 | 2 | 1 | 1 | 1 | 1 | positive | positive |
|  | 6 | -44.6 | 13.79 | 3300 | 12.1 | 0 | 2 | 2 | 2 | 2 | 1 | 1 | 1 | 1 | 1 | 1 | positive | negative |
|  | 7 | -30.4 | 4.52 | 4100 | 33.5 | 0 | 1 | 1 | 1 | 1 | 1 | 1 | 1 | 1 | 1 | 1 | positive | negative |
|  | 8 | -64.4 | 16.83 | 638.6 | 52.5 | 0 | 1 | 1 | 1 | 1 | 1 | 1 | 1 | 1 | 1 | 1 | positive | negative |
|  | 9 | -41.0 | 14.55 | 2300 | 10.4 | - | - | - | - | - | - | - | - | - | - | - | negative | negative |
|  | 10 | -57.4 | 11.17 | 3200 | 30.6 | 0 | 2 | 2 | 2 | 1 | 1 | 1 | 1 | 1 | 1 | 1 | positive | negative |
|  | 11 | -22.7 | 19.98 | 342 | 22.1 | 0 | 0 | 0 | 1 | 1 | 1 | 1 | 1 | 1 | 1 | 1 | positive | positive |
|  | 12 | -35.2 | 11.6 | 4100 | 13 | 0 | 0 | 0 | 0 | 1 | 1 | 1 | 1 | 1 | 1 | 1 | positive | negative |
|  | 13 | -39.1 | 10.7 | 2000 | 14.1 | 0 | 0 | 0 | 1 | 1 | 1 | 1 | 1 | 1 | 1 | 1 | positive | positive |
|  | 14 | -45.9 | 10.2 | 585.9 | 20.9 | 1 | 2 | 2 | 1 | 1 | 1 | 1 | 1 | 1 | 1 | 1 | positive | positive |
|  | 15 | -50.1 | 17.48 | 797.1 | 22.1 | 0 | 1 | 2 | 1 | 1 | 1 | 1 | 1 | 1 | 1 | 1 | positive | negative |
|  | AV | -44.9 | 15.9 | 3010.9 | 25.9 | 0.1 | 2.6 | 2.4 | 2.4 | 1.4 | 1.1 | 1.1 | 1.0 | 1.0 | 1.0 | 1.0 | 14ea / 15ea | 7ea / 15ea |
|  | SD | 12.8 | 6.1 | 3881.8 | 12.7 | 0.4 | 3.7 | 4.3 | 4.5 | 1.1 | 0.4 | 0.3 | 0.0 | 0.0 | 0.0 | 0.0 | 93.3 | 46.7 |
|  | SE | 3.3 | 1.6 | 1002.3 | 3.3 | 0.1 | 1.0 | 1.1 | 1.2 | 0.3 | 0.1 | 0.1 | 0.0 | 0.0 | 0.0 | 0.0 |  |  |

**Supplemental Figures with Figure Legends**


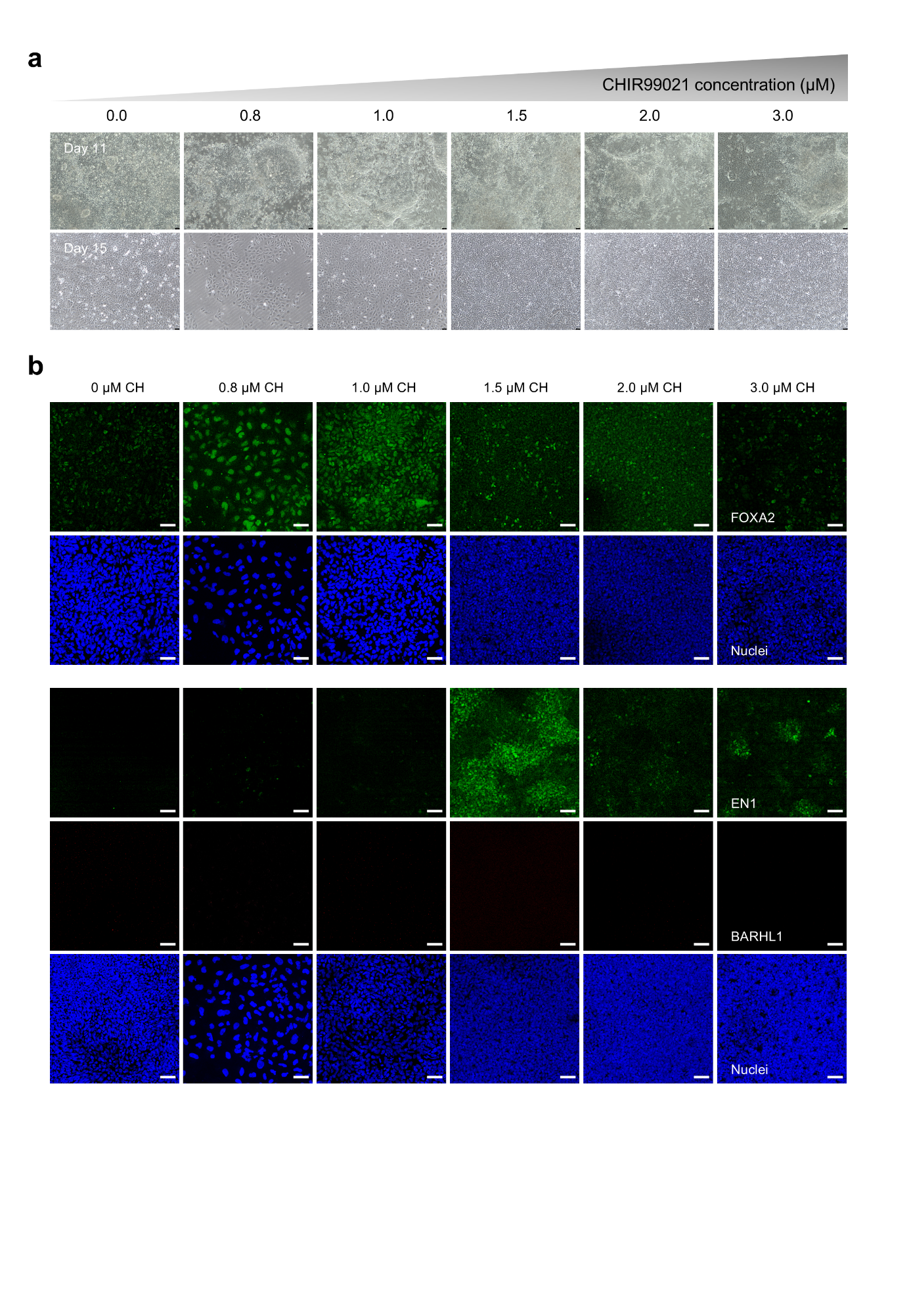


**Figure S1.  Ventral midbrain (VM) patterning of porcine embryonic stem cells according to the concentration of CHIR99021 in 2D condition.** (a) Morphological changes during differentiation on day 11 and day 15. Scale bar, 50 μm. (b) Immunostaining at day 16 for FOXA2, EN1, and BARHL1. Scale bar, 20 μm.


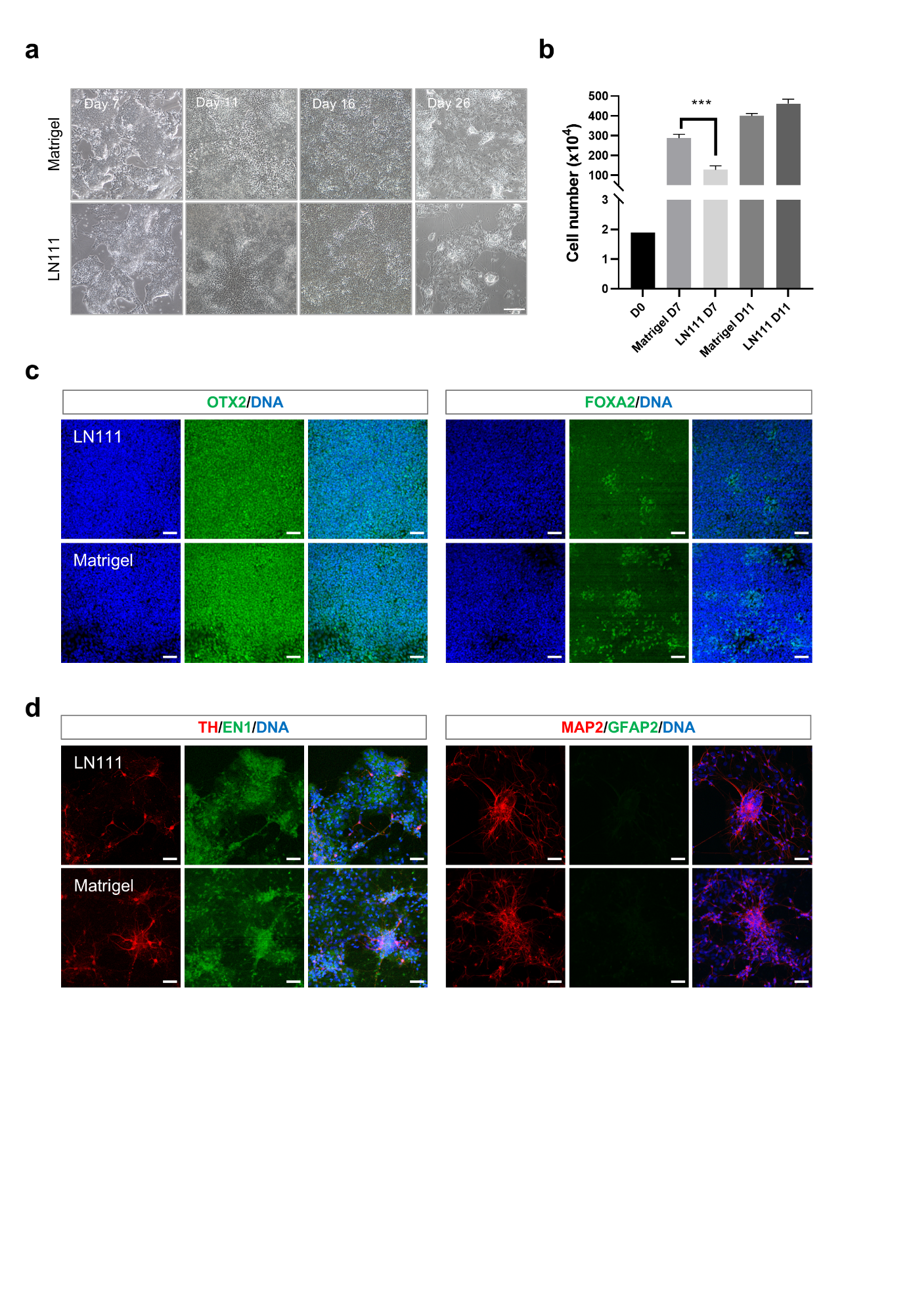


**Figure S2. Induction of porcine floor plate progenitors and neurogenesis using LN111 matrix.** (a) Phenotypic overview of cultures at different differentiation stages. Scale bar, 200 μm. (b) Cell yield by Matrigel or LN111 at day 7 and day 11 (n = 3 independent experiments). *** p < 0.001 (c) Immunostaining of OTX2+ cells and FOXA2+ cells at day 16. Scale bar, 200 μm. (d) Immnunostaining of TH+/EN1+ cells and MAP2+/GFAP2+ cells at day 26. Scale bar, 200 μm.


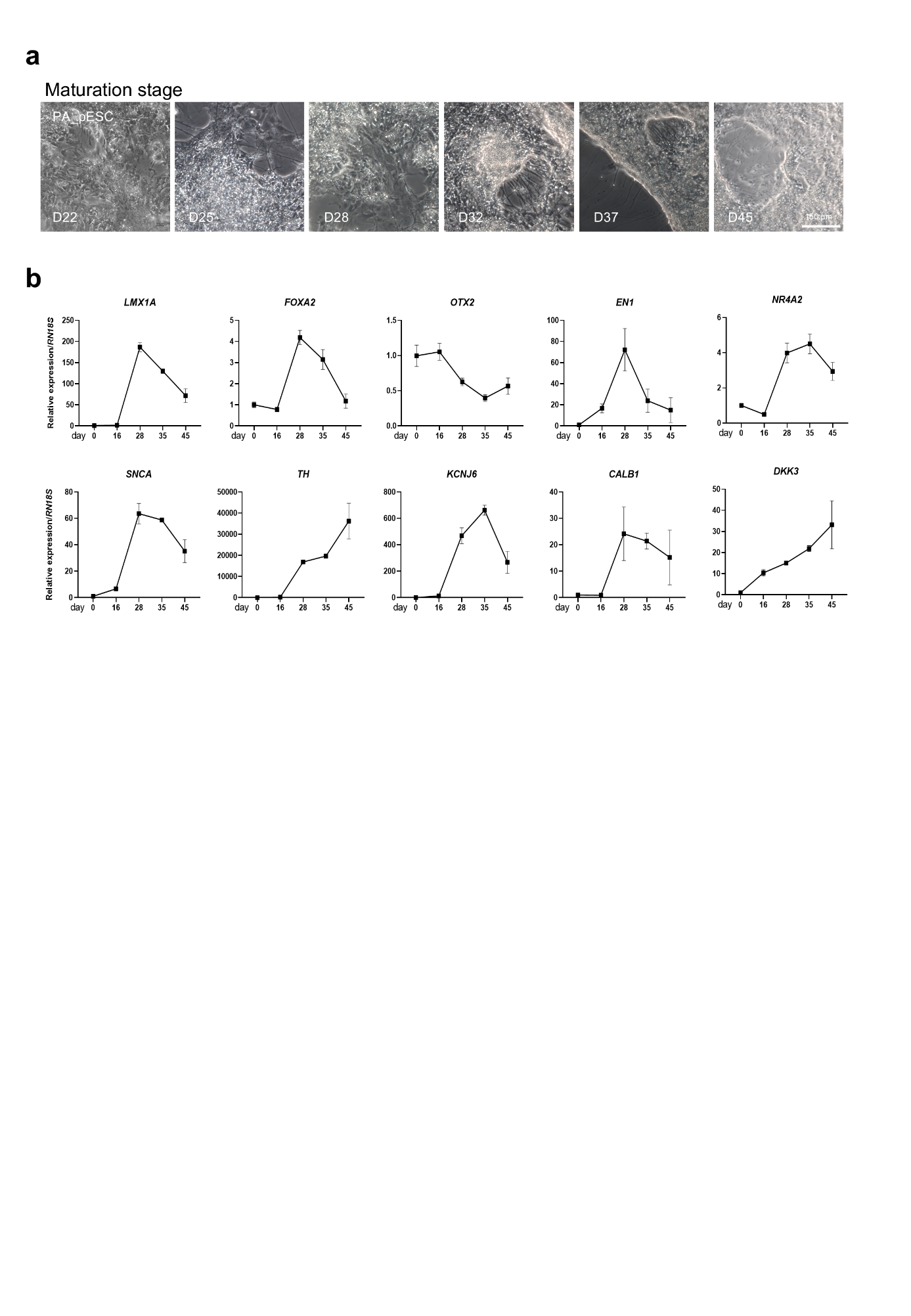


**Figure S3. Terminal differentiation of VM progenitors derived from PA-pESC.** (a) Morphological changes of PA-pESC at different differentiation stages. (b) RT-qPCR analysis during terminal differentiation of VM progenitors


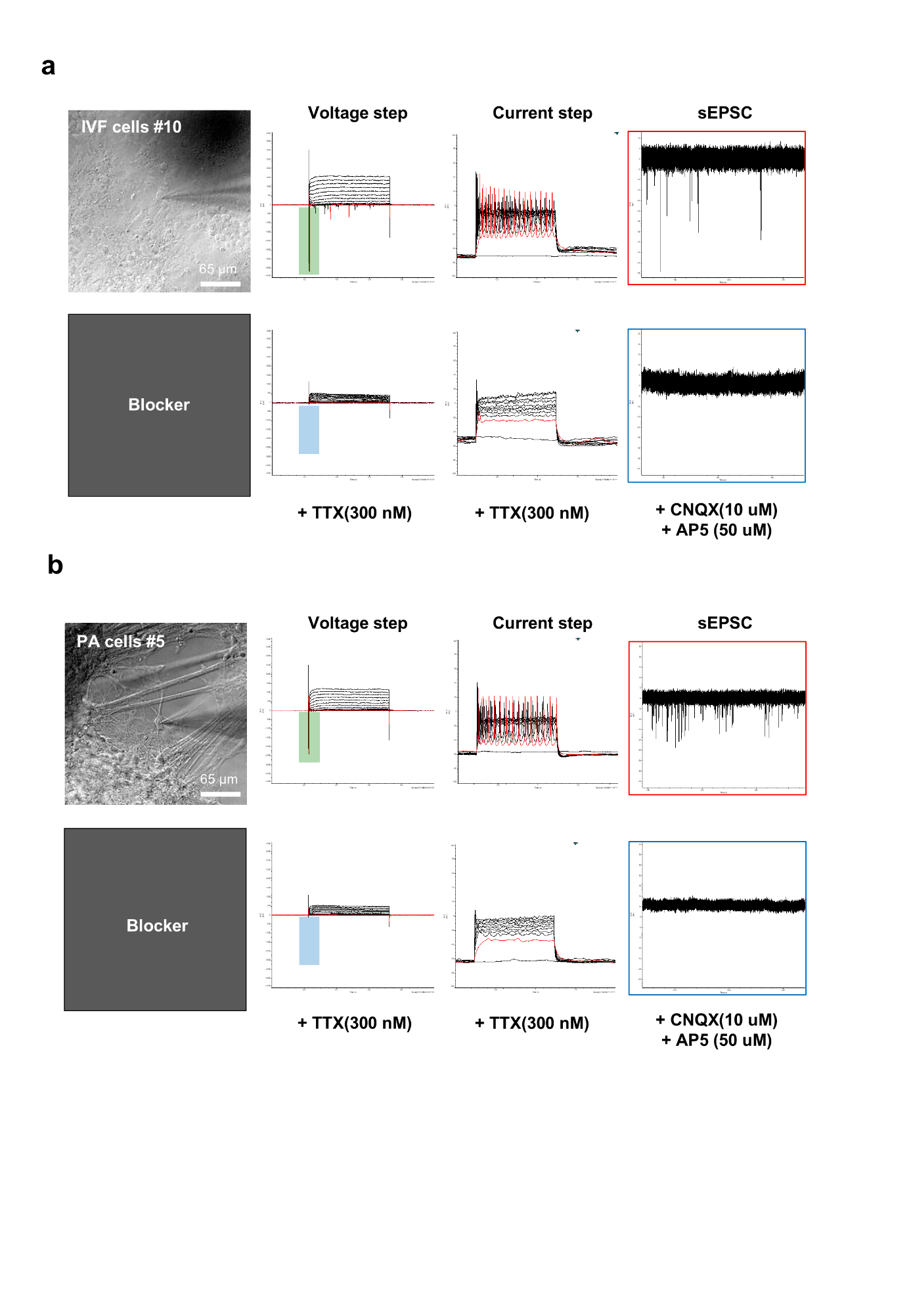


**Figure S4. spontaneous excitatory post-synaptic currents (sEPSC) detection.** Whole-cell patch-clamp recordings of IVF-derived (a) and PA-derived (b) neurons. sEPSCs were recorded for 5 min at –60 mV using a gap-free protocol under three conditions: control, with TTX (300 nM; voltage-gated sodium channel blocker), and with CNQX (10 μM; AMPA receptor antagonist) plus AP5 (50 μM; NMDA receptor antagonist).


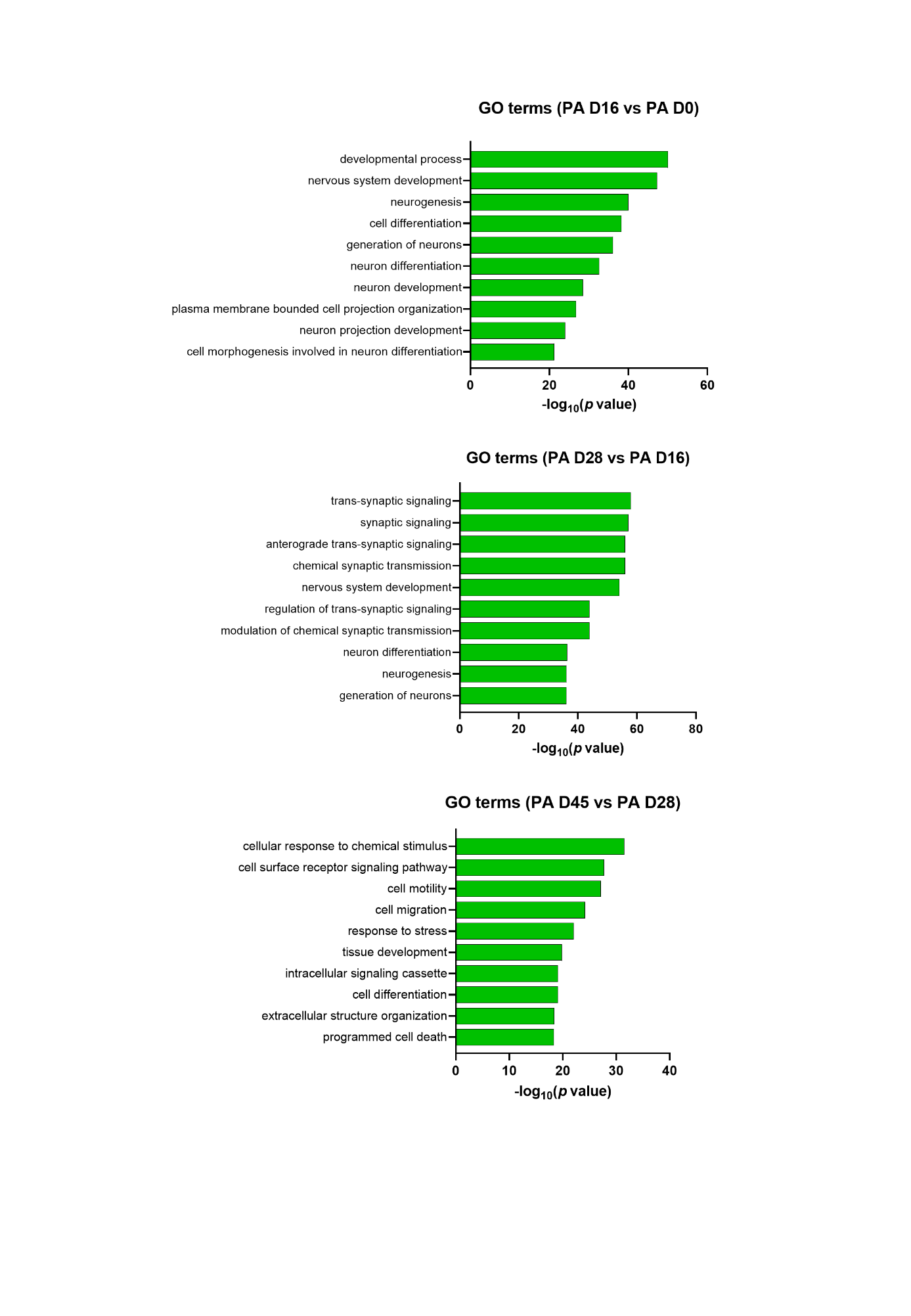


**Figure S5. Gene ontology (GO) enrichment analysis of differentially expressed genes in PA-derived cells.** GO enrichment analysis of differentially expressed genes (DEGs) in PA-derived cells between D0 and D16, D16 and D28, and D28 and D45 samples, performed using the same analysis pipeline as in Figure 5C.


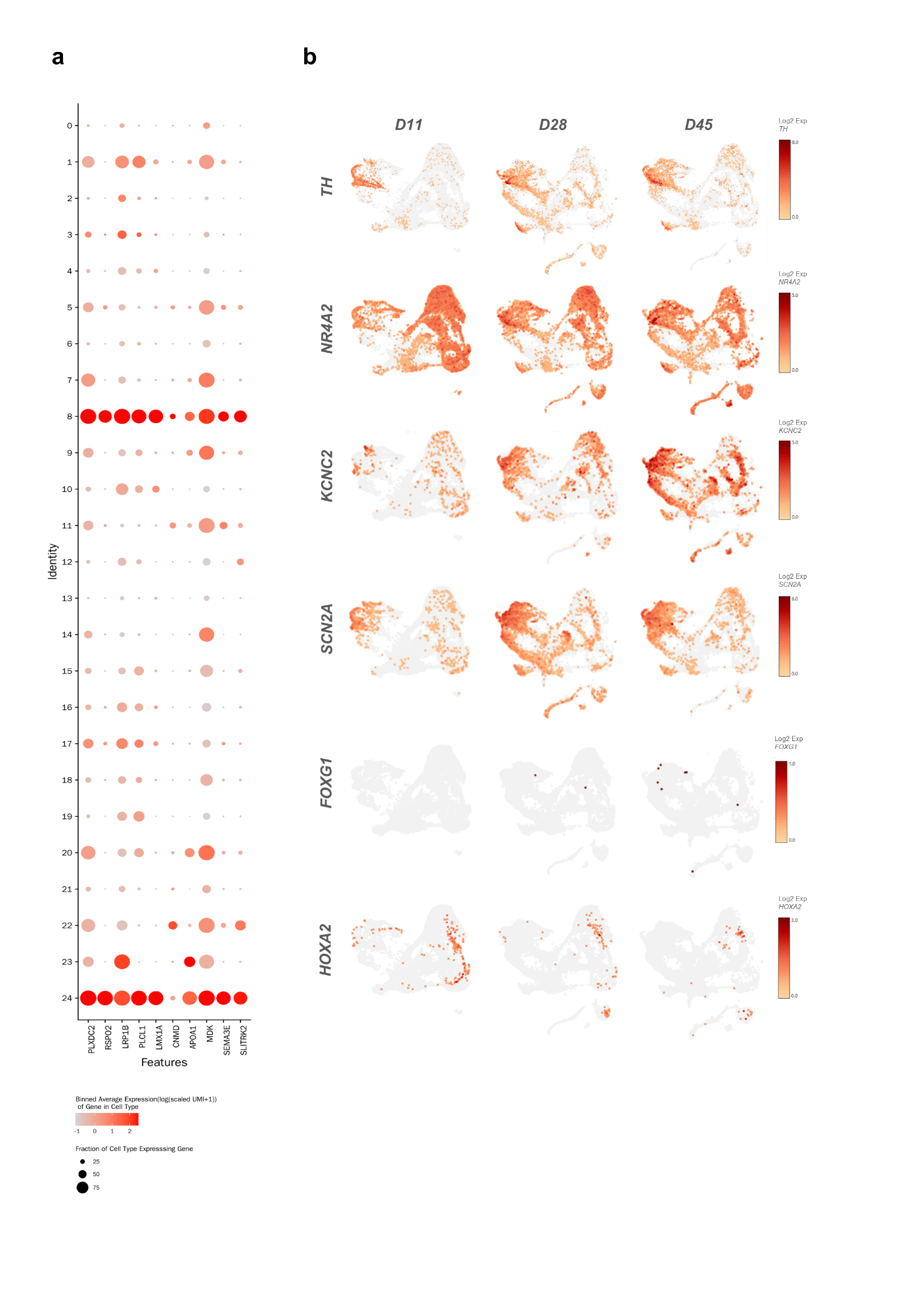


**Figure S6. Cluster-specific and temporal expression patterns of dopaminergic neuron markers in pigMLOs. (a)** Dot plot showing the expression levels and fraction of cells expressing dopaminergic lineage markers across identified clusters at day 45. **(b)** UMAP feature plots displaying expression of dopaminergic markers (TH, NR4A2, KCNC2, SCN2A) and regional identity markers (FOXG1 for forebrain, HOXA2 for hindbrain) at days 11, 28, and 45.
